# Effects of Tai Chi Qigong on sleep quality in older male cancer survivors: A randomized controlled clinical trial

**DOI:** 10.61373/bm026a.0063

**Published:** 2026-09-08

**Authors:** Evelyn Arana-Chicas, Michael R. Irwin, Cindy K. Blair, Jinghua An, Samuel Tundealao, Yong Lin, Shou-En Lu, Chunxia Chen, Biren Saraiya, Dolores Guest, Wadih Arap, Emily Heidt, Anita Y. Kinney

**Affiliations:** 1 Rutgers Cancer Institute, New Brunswick, NJ 08901; 2 Rutgers Cancer Institute, Newark, NJ 07103; 3 Department of Psychiatry, David Geffen School of Medicine, University of California, Los Angeles, CA 90095; 4 Cousins Center for Psychoneuroimmunology, Semel Institute for Neurosciences, University of California, Los Angeles, CA 90095; 5 University of New Mexico Comprehensive Cancer Center, Albuquerque, NM 87102; 6 Department of Internal Medicine, University of New Mexico, Albuquerque, NM 87102; 7 School of Nursing, Indiana University, Indianapolis, IN; 8 Department of Biostatistics and Epidemiology, Rutgers University School of Public Health, Piscataway, NJ 08901; 9 Division of Medical Oncology, Section of Solid Tumors, Robert Wood Johnson Medical School, Rutgers University, New Brunswick, NJ 08901; 10 Division of Hematology/Oncology, Department of Medicine, Rutgers New Jersey Medical School, Newark, NJ 07103

**Keywords:** Cancer, complementary therapy, mind-body therapies, qigong, sleep, tai chi

## Abstract

To compare the efficacy of Tai Chi and Qigong (TCQ) versus exercise intensity-matched (EIM) and usual care (UC) on sleep quality in older male cancer survivors. We conducted a three-arm randomized controlled trial among male cancer survivors aged 55 years and older. Participants were randomized to UC or one of two group-based interventions (TCQ or EIM), delivered twice weekly for 12 weeks with follow-up through 12 months. Sleep quality was assessed using the Pittsburgh Sleep Quality Index (PSQI). Linear contrasts estimated within-arm changes and between-arm differences with 95% confidence intervals (CIs). A total of 113 participants (mean age 69.1 (SD = 7 years); 77.0% White; 88.5% non-Hispanic) were enrolled. At 3-months post-intervention, between-arm differences in sleep quality were not significant (*p* > 0.05). The EIM arm showed significant within-arm improvements in sleep quality at the 6-week midpoint (*p* = 0.004), 1-week post-intervention (*p* = 0.048), and 3 months post-intervention (*p* = 0.011) from baseline. The TCQ arm showed nonsignificant trends toward within-arm improvement in sleep quality at the same time points. No significant effects were observed at 12 months. The EIM arm significantly improved sleep efficiency at 3 months compared with TCQ (*p* = 0.04). By 12 months, 62% of TCQ participants achieved clinical remission of sleep disturbance (PSQI ≤5), compared with 47% in the EIM arm and 43% in the UC arm (*p* > 0.05). Attendance was similar across TCQ (78.4%) and EIM (76.8%). TCQ and EIM produced modest short-term improvements in sleep quality among older male survivors.

## Introduction

Sleep disturbances are among the most prevalent and persistent symptoms reported by cancer survivors, affecting up to 60 percent of individuals after completing primary cancer treatment (1, 2). These disturbances, which include difficulty falling asleep and/or maintaining sleep, excessive daytime sleepiness, movement- or breathing-related disorders, and parasomnias, are associated with increased risks of morbidity and mortality because they can negatively influence treatment adherence and overall health and well-being (3, 4). Despite their high prevalence and clinical significance, sleep disturbances in survivorship care are often underrecognized and inadequately managed. They are frequently dismissed as inevitable consequences of cancer treatment or aging (5). Among older cancer survivors, sleep disturbances may arise from reduced physical activity, certain medications, persistent or treatment-related pain, psychosocial stressors, and coexisting health conditions (6).

There is considerable evidence supporting the use of non-pharmaceutical strategies to manage sleep problems. Exercise and mindfulness-based interventions have been shown to alleviate sleep disturbances and improve associated symptoms such as fatigue and distress (7–9). The American Academy of Sleep Medicine recommends cognitive-behavioral therapy for insomnia (CBT-I) as the first-line treatment for sleep disorders in cancer survivors (10); however, it is not generally included in survivorship care plans (11). Uptake remains limited due to barriers such as high costs (12) and provider shortages (13), and it may not be well-received by everyone (13, 14). Moreover, most research evaluating behavioral interventions for insomnia has focused on women with breast cancer (15, 16), creating a critical evidence gap for male cancer survivors. Few studies have examined interventions for sleep disturbance in men, and even fewer in older men (17).

Mindfulness-based practices that incorporate physical movement, relaxation, and meditation have shown promise for promoting sleep quality and overall physiological and mental well-being (8, 9, 18, 19). These practices are grounded in the Biopsychosocial Model, which posits that physical, psychological, and social factors interact to influence health (20). Tai Chi and Qigong (TCQ), both meditative movements originating in traditional Chinese medicine, combine gentle postures, deep breathing, and meditation to cultivate relaxation and balance. As a low-intensity exercise, TCQ is particularly suitable for older adults and individuals with limited mobility or chronic fatigue (21).

A growing body of research supports the use of tai chi and related mind-body interventions to improve sleep quality in older adults with chronic illnesses (22–24). Evidence in cancer survivors similarly demonstrates the benefits of tai chi on sleep outcomes (25–27). In breast cancer survivors, Irwin et al. reported that Tai Chi was statistically noninferior to Cognitive Behavioral Therapy for Insomnia (CBT-I) in women with breast cancer (28). Moreover, Mustian *et al*. found that yoga significantly improved sleep quality in solid-tumor cancer survivors, including older adults (18). Garland *et al*. likewise found that mindfulness produced clinically meaningful improvements in insomnia among cancer survivors, although CBT-I yielded faster and more durable effects (29). Extending these findings, Siu *et al*. found that tai chi was noninferior to CBT-I in middle-aged and older adults at 12-month follow-up, supporting its role in the long-term management of chronic insomnia (30). A separate study by Siu *et al*. also found no significant differences in sleep parameters between tai chi and exercise in older adults with insomnia (24). In a randomized controlled trial by Nguyen and Kruse, Tai Chi was consistently associated with improved subjective sleep quality in older adults (31).

Despite these benefits of Tai Chi and related mind-body interventions on sleep quality in cancer survivors and older adults, little is known about whether these benefits extend to male cancer survivors. To date, only one small pilot randomized controlled trial (RCT) among men with prostate cancer undergoing radiotherapy found that TCQ participants had longer sleep duration and improved sleep quality compared to those in light exercise and wait-list control groups (32). The limited research on the effects of TCQ on sleep quality among male cancer survivors highlights the critical need for further investigations into this area of research.

Building on our previous work with our Health Recovery and Empowerment Outcomes (HERO) randomized controlled trial (RCT; 33, 34), this study examines the efficacy of a standardized TCQ intervention for reducing sleep disturbance compared to an exercise intensity-matched (EIM) intervention and usual care (UC) among older male cancer survivors. This study aimed to enhance understanding of TCQ’s role in improving sleep quality among older male cancer survivors—a population for whom effective behavioral sleep interventions remain underdeveloped and underutilized.

## Results

A total of 113 male cancer survivors were enrolled in the study. Study enrollment, random-arm assignment, and retention data are presented in the CONSORT diagram of the primary outcomes manuscript (33). Table 1 reports participant demographic and clinical characteristics by intervention group. The mean age was 69.1 (±7.0) years. Most participants were White (77.0%), non-Hispanic (88.5%), and more than half held a bachelor’s degree (64%). The median number of years since the initial cancer diagnosis was four years (range: 0–24 years). The majority had a diagnosis of prostate cancer (87%).

Participants in both the TCQ and EIM groups demonstrated high average attendance rates across the 24 classes, with no statistically significant differences observed between the groups (TCQ [78.4%] and EIM [76.8%]). Treatment expectancy and credibility were similar between groups. Perceived exertion levels remained within the light exertion range throughout the intervention with no clinically meaningful differences (TCQ [9.8 ± 2.1] and EIM [10.9 ± 2.2]).

### Global sleep quality

At the 3-month assessment, between-arm differences in global sleep quality were not statistically significant (Table 2). Overall, no statistically significant between-arm differences were observed at any time point across any pairwise comparisons (i.e., TCQ vs. EIM, TCQ vs. UC, EIM vs. UC). The EIM arm showed statistically significant within-arm improvements in global sleep quality at the 6-week midpoint assessment (mean change = −1.20; 95% CI [−2.02, −0.39]), 1-week post-intervention (mean change = −0.82, 95% CI [−1.63, −0.01]), and at 3 months post-intervention (mean change = v1.04, 95% CI [−1.84, −0.24]; Table 2) from baseline. TCQ showed nonsignificant trends toward within-arm improvement in global sleep quality at these same timepoints: 6-week midpoint ( −0.70, 95% CI [−1.44, 0.04]), 1-week post-intervention (−0.65, 95% CI [−1.39, 0.08]), and 3 months post-intervention (mean change = −0.59, 95% CI [−1.34, 0.16]; Table 2) from baseline. These improvements were similarly reflected in two sensitivity analyses: one employing multiple imputation (Supplemental Table S1) and the other focusing exclusively on participants with prostate cancer (*n* = 98, Supplemental Table S2). Per-protocol analyses showed similar improvements in global sleep quality (Supplemental Tables S3 and S4).

**Figure 1.**
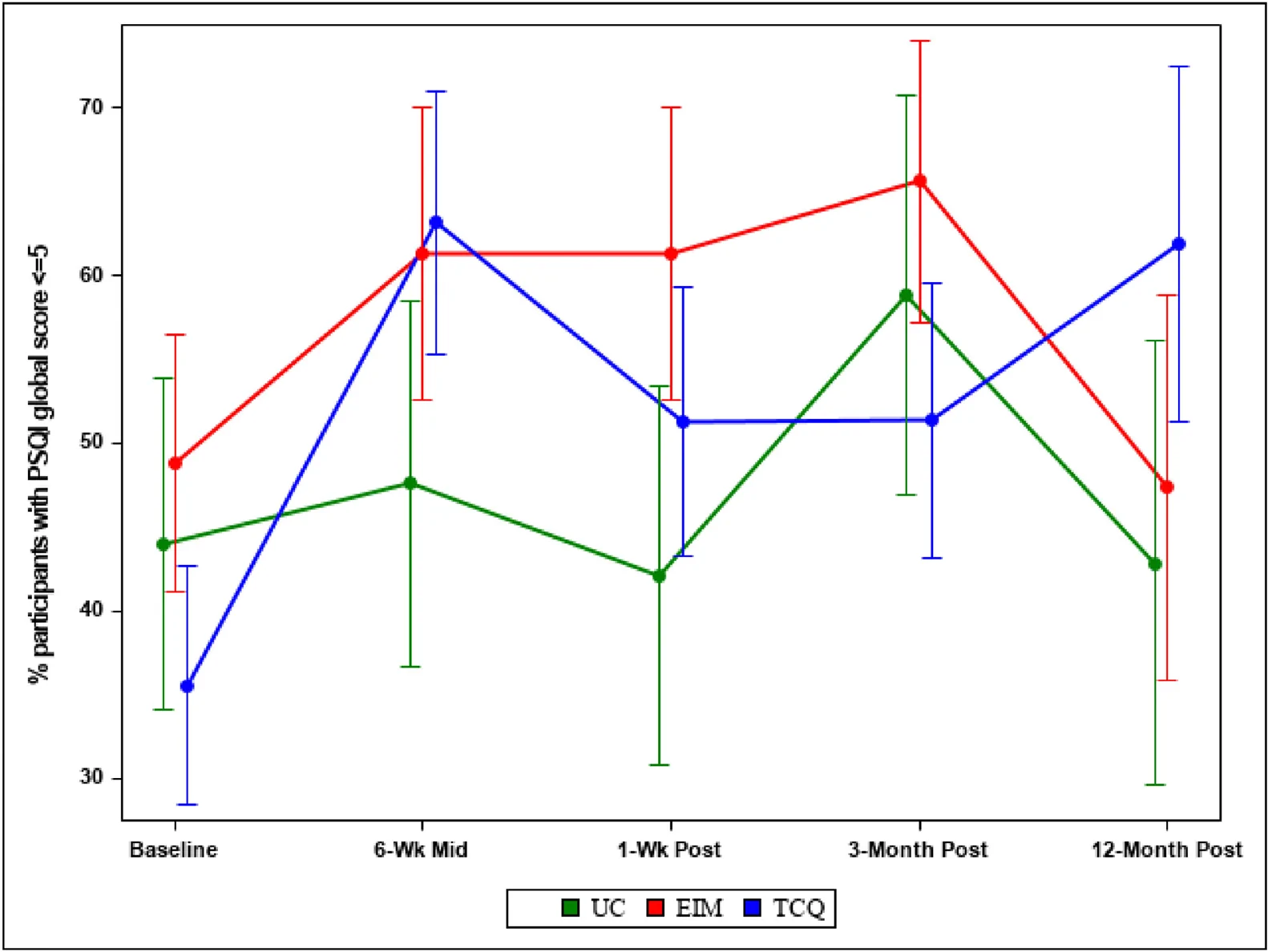
Proportion of participants in each study arm who achieved clinical remission of sleep disturbance (PSQI score ≤5) at each assessment time point.

Figure 1 presents the proportion of participants in each study arm who achieved clinical remission of global sleep disturbance (PSQI score ≤5) at each assessment time point. At baseline, remission rates were similar between groups, with only small differences observed between TCQ, EIM, and UC participants. Over the course of the intervention, remission proportions increased in both active treatment arms, whereas the UC group showed more modest change. At 3-months post-intervention, 51% of TCQ participants had clinical sleep remission, compared with 65% in the EIM arm, and 58% in the UC arm (TCQ vs UC *p*-value = 0.61; TCQ vs EIM *p*-value = 0.23; EIM vs UC *p*-value = 0.64). By 12 months post-intervention, 62% of TCQ participants met clinical sleep remission criteria (PSQI score ≤5), compared with 47% in the EIM and 43% in the UC groups, although these changes were not significant (TCQ vs UC *p*-value = 0.68; TCQ vs EIM *p*-value = 0.99; EIM vs UC *p*-value = 0.67).

### PSQI subscales

Consistent with the primary outcome, most PSQI subscales showed no significant between-arm differences. Within-arm changes in daytime dysfunction, sleep disturbances, sleep duration, sleep latency, subjective sleep quality, and use of sleep medication were generally nonsignificant or limited to isolated trends (Table 2). In contrast, the sleep efficiency subscale demonstrated a more robust pattern: the EIM arm showed statistically significant improvements at 3 months post-intervention (−0.46, 95% CI [−0.76, −0.15]), resulting in a significant between-arm difference favoring EIM over TCQ (0.44, 95% CI [0.02, 0.86]). These patterns were consistent in sensitivity analyses by using multiple imputation and in the prostate cancer subsample, as well as in per-protocol analyses (Supplemental Tables S1–S4).

An exploratory sensitivity analysis examining intervention delivery format (in-person versus hybrid) showed similar patterns of sleep outcomes across groups, suggesting that the primary findings were not substantially influenced by the mode of delivery (Supplemental Tables S5 and S6).

## Discussion

In this secondary analysis of older male cancer survivors aged 55 years and older, there were no statistically significant between-arm differences in global sleep quality across any pairwise comparisons (TCQ vs. EIM, TCQ vs. UC, or EIM vs. UC) at the 3-month primary endpoint. Within groups, the EIM arm demonstrated small but statistically significant improvements from baseline at the 6-week mid-intervention, 1-week post-intervention, and 3-months post-intervention from baseline, whereas TCQ showed non-significant trends toward improvement at these same time points. All changes were below the 3-point change threshold for clinically meaningful differences (35). Sensitivity and per-protocol analyses yielded similar findings. Despite limited effects on mean PSQI scores, a greater (but non-significant) proportion of TCQ participants achieved clinical remission of sleep disturbance by 12 months (62% vs. 47% EIM and 43% UC). Consistent with the primary outcome, most PSQI subscales showed minimal change, except for sleep efficiency, which improved significantly in the EIM arm, resulting in a modest between-arm difference favoring EIM over TCQ.

These findings are generally consistent with prior research on TCQ and related mind–body movement interventions in cancer survivors, which have reported modest but meaningful improvements in sleep outcomes (18, 28, 29, 32), except for one study that found no significant benefit (36). Although no significant between-group differences were observed, the pattern of findings showing high rates of PSQI remission at 12 months in the TCQ arm and short-term improvements in the EIM arm aligns with work by Siu *et al*. (24). However, these observations are exploratory and should be interpreted with caution, given the absence of statistically significant between-group differences and the limited statistical power of the study. Differences across studies may reflect variability in TCQ style, exercise intensity, session frequency, and total dosage, as well as heterogeneity in baseline sleep disturbance and cancer type. Importantly, intent-to-treat and per-protocol analyses in the present study yielded similar patterns, suggesting that the observed effects were not driven solely by adherence or differential dropout. Together, these results contribute to a growing body of evidence indicating that gentle movement-based practices and conventional exercise may offer a feasible nonpharmacologic option for improving sleep in cancer survivorship, while highlighting the need for greater standardization of intervention delivery and identification of patient subgroups most likely to benefit.

An additional consideration is that participants were enrolled based on clinically significant fatigue rather than sleep disturbance. Because cancer-related fatigue and sleep disturbance frequently co-occur and may share common behavioral and biological mechanisms (37, 38), the study population was likely enriched for survivors experiencing both symptoms. Consequently, the observed sleep responses may not generalize to cancer survivors whose primary concern is sleep disturbance without significant fatigue.

This research has several limitations and strengths. The analyses were underpowered to detect between-arm differences due to the small sample size. The small sample size also limited our ability to conduct additional subgroup analysis. Generalizability was limited by the study population’s demographic homogeneity, which consisted primarily of college-educated, non-Hispanic White participants. Given documented disparities in sleep quality and access to supportive care services (39), future research should prioritize recruiting diverse populations. Tailoring TCQ interventions to specific cultural contexts may increase acceptability and efficacy, especially among underrepresented Hispanic and Black cancer survivors (40). Another limitation is that the sample consisted predominantly of prostate cancer survivors (87%). Although exploratory analyses suggested generally similar patterns of sleep outcomes among participants with other cancer types, the number of non-prostate cancer survivors was small (*n* = 15). In addition, by chance, no men without a diagnosis of prostate cancer were randomized to the usual-care arm, limiting comparisons across all three study groups. Therefore, the applicability of these findings to survivors with other types of cancer remains uncertain and should be evaluated in future studies with more diverse cancer populations. Furthermore, because sleep outcomes were assessed solely via self-reported PSQI measures, conclusions about the maintenance of sleep improvements over time should be interpreted with caution. Future research should incorporate objective sleep metrics, such as actigraphy or wearable devices, to more accurately characterize sleep patterns. Moreover, interpretation of the PSQI subscale findings should be made with caution, as multiple comparisons were performed and these analyses were exploratory. Consequently, the statistically significant between-group difference in sleep efficiency may reflect a chance finding and should be confirmed in future, adequately powered studies. Because relatively few participants were in the oldest age groups (age 80+ years), our ability to evaluate outcomes among older survivors was limited. Home-practice logs were collected but completed inconsistently, limiting our ability to examine adherence or to evaluate whether differences in home engagement contributed to the observed intervention effects. Limited COVID-19 reporting reduced our ability to evaluate pandemic-related effects. However, exploratory sensitivity analyses examining delivery format (in-person vs. hybrid) demonstrated similar patterns of sleep outcomes across intervention groups, suggesting that the overall findings were not appreciably influenced by mode of intervention delivery. Strengths of the study included a rigorous three-arm design, inclusion of active and non-active control groups, a specific focus on male cancer survivors, a population often underrepresented in mind-body exercise research. Additional strengths include strong class attendance, comparable exercise intensity across groups, and similar perceptions of treatment credibility.

In conclusion, TCQ and EIM showed modest benefits for sleep among male cancer survivors, with generally small improvements and limited clinically meaningful differences. While short-term improvements were observed, the overall pattern suggests that both TCQ and EIM may yield comparable sleep benefits, and additional work is needed to clarify potential differences in longer-term outcomes. Future adequately powered and demographically diverse trials are needed to confirm these effects and guide the development of clinical recommendations for managing sleep disturbance among older male cancer survivors.

## Methods

### Study design

This is a primary analysis of a secondary outcome of a parallel-group, three-arm RCT; the study protocol and primary patient-reported outcome (cancer-related fatigue) results have been published previously (33, 34). This RCT was approved by the Institutional Review Boards at Rutgers University and the University of New Mexico. Participants were recruited from the Rutgers Cancer Institute, the University of New Mexico Comprehensive Cancer Center, and surrounding community locations in New Brunswick, New Jersey, and Albuquerque, New Mexico. Upon providing informed consent and completing baseline assessments, participants were randomized in a 2:2:1 ratio to one of three arms: 1) TCQ, 2) EIM, or 3) Usual care.

Research staff enrolled participants and communicated group assignments. Men in the TCQ and EIM groups were not informed whether they were assigned to the experimental or control condition. The terms “Tai Chi” and “Qigong” were not used in study materials. Instead, we labeled the intervention groups as follows: the TCQ intervention as body-mind training (BMT), and the EIM condition as body training (BT). This strategy aimed to limit potential bias by reducing participants’ expectations and minimizing perceived distinctions among the study arms. Both interventions were delivered concurrently over 12 weeks. Follow-up assessments were administered at 6 weeks mid-intervention, 1-week post-intervention, 3 months, and 12 months post-intervention. Recruitment took place between June 2017 and November 2021, with intervention delivery spanning August 2017 and February 2022. The Clinicaltrials.gov identifier is NCT03345563.

### Participants

Eligibility criteria included being male, age 55 years or older, and having a confirmed diagnosis of localized or regional stages of colorectal, small intestine, lung, thyroid, soft tissue, kidney, non-muscle invasive bladder, or renal pelvis, or prostate cancer (including men with biochemical disease (elevated prostate-specific antigen). Additional criteria included remission or stable disease and completion of primary cancer treatment, except for hormone manipulation therapy. Participants receiving hormone manipulation therapy were required to have been on therapy for at least 4 months. Eligible individuals also needed to score below 13 on the SF36 Vitality subscale (41) or above 9 on the PROMIS Fatigue scale (42) and engage in fewer than 150 minutes of moderate-to-vigorous physical activity per week over the previous 3 months, demonstrate English language proficiency, and have medical clearance for participation in light to moderate physical activity. Exclusion criteria included having a Patient Health Questionnaire-9 (PHQ-9) score greater than 11 (indicating moderate-to-severe depression); 2) Karnofsky Performance Status below 51%; 3) reporting engagement in a mindfulness-based intervention(s; ≥2 sessions/week for ≥2 months within the past year; and/or 4) currently receiving cancer treatment other than ADT.

### Interventions

#### Tai Chi Qigong (TCQ)

The standardized intervention has been described previously (33, 34). TCQ classes were delivered by a Qigong Master or a certified instructor trained in the study protocol. The 12-week program consisted of two 60-minute sessions per week and incorporated 19 exercises, including deep breathing, flowing movements, synchronized meditative movements, postural sequences, and guided relaxation. To ensure safety while gradually intensifying the intervention, movements that could be performed standing or seated (such as the rocking chair or Tai Chi ruler) were selected. Classes were complemented with home practice materials, including an instructional DVD and handouts outlining specific poses to support independent practice. Participants were encouraged to practice at home for at least 30 minutes a day, three days a week, throughout the intervention and 12-month follow-up period.

#### Exercise intensity-matched (EIM) intervention

The EIM intervention paralleled TCQ in overall intensity, session duration, group interaction, cardiopulmonary demand, and delivery format. It incorporated controlled stretching, as well as static and eccentric repetitive exercises, developed by two exercise physiologists to approximate TCQ cardiopulmonary exertion, exercise intensity, posture, and muscle activation (34). As with TCQ participants, EIM participants were provided with an instructional DVD and handouts illustrating specific movements and were encouraged to engage in home practice for at least 3 days/week, for a minimum of 30 minutes.

Class sessions for both interventions were delivered in person until the onset of the COVID-19 pandemic in March 2020, after which sessions transitioned to synchronous hybrid group formats. In September 2020, participants were given the option to attend sessions either in person or in a hybrid format.

#### Usual care

Participants in the UC arm received care as usual and did not participate in classes. These participants completed the same assessments as participants in the intervention groups.

### Data collection and measures

Baseline sociodemographic and clinical information (i.e., cancer diagnosis, cancer stage, and hormone therapy status) was obtained through self-report and medical record abstraction.

#### Primary outcome for this secondary analysis

Sleep quality was assessed by using the Pittsburgh Sleep Quality Index (PSQI) global score from baseline to 3-months, while other outcomes included PSQI global score change at 6-week mid-intervention, 1-week post-intervention, and 12-months post-intervention, as well as changes in PSQI subscale scores at all time-points. The PSQI is a validated 19-item instrument with a global score (*α* = 0.83; score range, 0–21) (43) and seven subscales that reflect sleep quality: 1) subjective sleep quality, 2) sleep latency, 3) sleep duration, 4) sleep efficiency, 5) sleep disturbance, 6) use of sleep medication, and 7) daytime dysfunction. Higher scores reflect poorer sleep quality. A global score above 5 indicates clinically significant sleep disturbance, while a score of 5 or less indicates clinical remission or good sleep quality (43). A reduction of at least 3 points or more for the global score is considered clinically meaningful (35). To date, no clinically meaningful difference has been established for any individual PSQI subscale.

Treatment credibility and expectancy were assessed at three time points: during the first week of class, at the 6-week midpoint, and one week after the intervention concluded. A 6-item credibility/expectancy scale, adapted for group-based health interventions, was used to measure participants’ perceptions of the intervention’s plausibility and their expectations regarding potential benefits (44). The scale demonstrates strong internal consistency (*α* = 0.79–0.81) and excellent test-retest reliability.

The Borg scale, a widely used measure of perceived exertion, was administered to TCQ and EIM participants to monitor and guide exercise intensity (45). A research assistant attended each session to monitor intervention fidelity by using standardized checklists, and all sessions were judged adherent. Class attendance was also monitored.

### Statistical analyses

Statistical analyses followed the intent-to-treat principle. The primary outcome for these analyses was the PSQI global score change from baseline to 3-months, while secondary outcomes included PSQI global score change at 6-week mid-intervention, 1-week post-intervention, and 12-months post-intervention, as well as changes in PSQI subscale scores at all timepoints. Mixed-effects models were applied, with the intervention group, time, and the group-by-time interaction treated as fixed effects, and participants included as a random effect for repeated measures. Linear contrasts were used to estimate within-arm score changes and to compare them between groups, with 95% confidence intervals (CIs). Missing data were handled by using fully conditional specification multiple imputation, assuming data were missing at random (46). Perprotocol analyses were conducted among participants who completed assessments through the 6-week midpoint and 1-week post-intervention. Remission of sleep disturbance was evaluated across groups by using chi-square tests. The effect of the interventions in men with prostate cancer was analyzed as a sensitivity analysis. The percentage of participants with remission of sleep disturbance (PSQI score ≤5) was calculated, and the differences among the three intervention groups were tested at each time point separately by using the chi-square test. Statistical significance was defined as a two-sided *p*-value < 0.05. SAS version 9.4 (SAS Institute Inc., Cary, NC) was used to perform all analyses. Generalized estimating equations, marginal logistic regression analysis, and linear contrasts were used to compare the proportions of PSQI ≤5 points between treatment arms at each time point as well as the changes from each time point from the baseline within each treatment arm. Analyses did not control for covariates known to be associated with sleep quality, as randomization procedures ensured group equivalence on demographic, psychosocial, and medical factors (e.g., ADT, age, comorbidities, BMI, and sex). Additionally, an exploratory sensitivity analysis was conducted to examine whether patterns of change in sleep outcomes differed according to intervention delivery format (in-person versus hybrid).

Sample size determination and power were described previously (33, 34). A target of 123 subjects across three treatment groups (2:2:1 ratio) was determined to detect between-arm differences (Cohen’s *d* = 0.63 for TCQ vs. EIM, 0.78 for TCQ vs. UC) with 80% power and *α* = 0.025 (two-sided after Bonferroni adjustment) for each comparison. After adjusting for a 25% attrition rate, 166 participants were planned; however, COVID-19 disruptions limited enrollment to 113 (45, 43, and 25 participants for TCQ, EIM, and UC arms, respectively).

## Supplementary Material

Supplementary Tables

## Figures and Tables

**Table 1.** Sociodemographic and clinical characteristics of subjects by study Arm (*N* = 113)

Study arm	All (N = 113) n (%)	TCQ (N = 45) n (%)	EIM (N = 43) n (%)	UC (N = 25) n (%)	*P* value^a^

Age (mean, standard deviation [SD])	69.1 (7.0)	69.4 (7.0)	68.3 (7.2)	70.3 (6.9)	0.51
Years since initial diagnosis (mean, SD)	5.9 (5.2)	5.9 (5.9)	6.1 (4.5)	5.7 (5.1)	0.94
Body mass index (mean, SD)	30.3 (6.5)	31.2 (8.1)	29.6 (5.4)	29.7 (4.8)	0.47
Hispanic ethnicity					0.16
No	100 (88.5)	37 (82.2)	41 (95.3)	22 (88.0)	
Yes	13 (11.5)	8 (17.8)	2 (4.7)	3 (12.0)	
Race					0.86
White	87 (77.0)	33 (73.3)	33 (76.7)	21 (84.0)	
Black	14 (12.4)	6 (13.3)	6 (14.0)	2 (8.0)	
Other^b^	12 (10.6)	6 (13.3)	4 (9.3)	2 (8.0)	
Marital status					0.78
Single/divorced/separated/widowed	26 (23.0)	9 (20.0)	10 (23.3)	7 (28.0)	
Married/domestic partnership	86 (76.1)	35 (77.8)	33 (76.7)	18 (72.0)	
Missing	1 (0.9)	1 (2.2)	0	0	
Education level					0.12
Less than high school/high school grad/GED	16 (14.2)	11 (24.4)	4 (9.3)	1 (4.0)	
Some college/assoc. degree/vocational school	32 (28.6)	13 (28.9)	11 (25.6)	8 (32.0)	
Bachelor’s degree or higher	64 (57.1)	21 (46.7)	27 (62.8)	16 (64.0)	
Missing	1 (0.9)	0	1 (2.3)	0	
Income					0.69
<$30,000	11 (9.7)	5 (11.1)	5 (11.6)	1 (4.0)	
$30,000–$49,999	8 (7.1)	2 (4.4)	5 (11.6)	1 4.0)	
$50,000–$69,999	14 (12.4)	7 (15.6)	4 (9.3)	3 (12.0)	
$70,000 or more	58 (51.3)	21 (46.7)	23 (53.5)	14 (56.0)	
Missing	22 (19.5)	10 (22.2)	6 (14.0)	6 (24.0)	
Health insurance					0.54
No	3 (2.6)	2 (4.4)	1 (2.3)	0	
Yes	109 (96.5)	43 (95.6)	42 (97.7)	24 (96.0)	
Missing	1 (0.9)	0	0	1 (4.0)	
Cancer diagnosis					0.12
Prostate only	87 (77.0)	36 (80.0)	30 (69.8)	21 (84.0)	
Prostate and other types of invasive cancer(s)	11 (9.7)	2 (4.4)	5 (11.6)	4 (16.0)	
Other cancer type(s)	15 (13.3)	7 (15.6)	8 (18.6)	0	
Stage of disease					0.30
Local	73 (64.6)	27 (60.0)	28 (65.1)	18 (72.0)	
Regional	10 (8.8)	2 (4.4)	6 (13.9)	2 (8.0)	
Distant/biochemical disease	30 (26.6)	16 (35.6)	9 (21.0)	5 (20.0)	
Currently taking hormone therapy					0.15
No	84 (74.3)	29 (64.4)	35 (81.4)	20 (80.0)	
Yes	29 (25.7)	16 (35.6)	8 (18.6)	5 (20.0)	
Comorbidities (at least one is present)					
Cardiometabolic^c^	75 (66.4)	32 (71.1)	28 (65.1)	15 (60.0)	0.63
Lung disease^d^	18 (16.0)	5 (11.1)	9 (20.9)	4 (16.0)	0.45
Neurological conditions^e^	10 (8.9)	4 (8.9)	4 (9.3)	2 (8.0)	1.0

TCQ, Tai Chi and Qigong; EIM, exercise intensity matched; UC, usual care; SD, standard deviation.

a Demographic and clinical characteristics of participants were compared among three arms using ANOVA and chi-square analysis.

b ‘Other’ races include: 2 American Native or Alaska Native, 1 Chinese, 1 Indian, 1 Filipino, 1multiracial, and 6 Hispanic participants did not specify their race.

c Cardiometabolic conditions included stroke, hypertension, diabetes, congestive heart failure, and peripheral vascular disease.

d Lung disease included chronic obstructive pulmonary disease, chronic bronchitis, and emphysema.

e Neurological conditions included connective tissue disease and hemiplegia.

**Table 2.** Changes in sleep from baseline: linear mixed model analysis for within and between-arm comparison (*N* = 113)

			6-week mid-intervention		1-week post intervention		3-month post-intervention		12-month post-intervention	
		Baseline Mean (SE)	Mean (SE)	Change from baseline MD (95% CI; *p*-value)	Mean (SE)	Change from baseline MD (95% CI; *p*-value)	Mean (SE)	Change from baseline MD (95% CI; *p*-value)	Mean (SE)	Change from baseline MD (95% CI; *p*-value)

PSQI	TCQ	6.47 (0.50)	5.77 (0.51)	−0.70 (−1.44, 0.04; 0.065)	5.82 (0.51)	−0.65 (−1.39, 0.08; 0.082)	5.88 (0.52)	−0.59 (−1.34, 0.16; 0.123)	6.24 (0.53)	−0.23 (−1.02, 0.56; 0.569)
	EIM	6.69 (0.51)	5.48 (0.55)	−1.20 (−2.02, −0.39; 0.004)	5.86 (0.55)	−0.82 (−1.63, −0.01; 0.048)	5.65 (0.54)	−1.04 (−1.84, −0.24; 0.011)	6.81 (0.55)	0.12 (−0.70, 0.94; 0.770)
	UC	6.24 (0.67)	5.91 (0.69)	−0.33 (−1.34, 0.67; 0.514)	5.60 (0.71)	−0.64 (−1.68, 0.40; 0.228)	5.30 (0.72)	−0.94 (−2.02, 0.14; 0.089)	5.99 (0.74)	−0.25 (−1.38, 0.87; 0.658)
	TCQ vs. UC			−0.36 (−1.61, 0.89; 0.567)		−0.02 (−1.29, 1.26; 0.980)		0.35 (−0.97, 1.67; 0.603)		−0.23 (−1.02, 0.56; 0.569)
	EIM vs. UC			−0.87 (−2.16, 0.42; 0.187)		−0.18 (−1.50, 1.14; 0.784)		−0.10 (−1.45, 1.25; 0.882)		0.12 (−0.70, 0.94; 0.770)
	TCQ vs. EIM			0.51 (−0.60, 1.61; 0.367)		0.17 (−0.93, 1.26; 0.764)		0.45 (−0.65, 1.55; 0.421)		−0.25 (−1.38, 0.87; 0.658)
psqi_daytime	TCQ	0.73 (0.10)	0.61 (0.10)	−0.12 (−0.32, 0.07; 0.216)	0.57 (0.10)	−0.16 (−0.36, 0.03; 0.102)	0.62 (0.11)	−0.10 (−0.31, 0.10; 0.303)	0.64 (0.11)	−0.09 (−0.30, 0.12; 0.422)
	EIM	0.74 (0.10)	0.71 (0.11)	−0.04 (−0.25, 0.18; 0.731)	0.70 (0.11)	−0.05 (−0.26, 0.16; 0.659)	0.74 (0.11)	−0.01 (−0.22, 0.20; 0.936)	0.85 (0.11)	0.11 (−0.11, 0.32; 0.332)
	UC	1.00 (0.13)	1.03 (0.14)	0.03 (−0.24, 0.29; 0.848)	0.91 (0.14)	−0.09 (−0.37, 0.18; 0.512)	0.89 (0.15)	−0.11 (−0.39, 0.18; 0.460)	0.89 (0.16)	−0.11 (−0.41, 0.18; 0.455)
	TCQ vs. UC			−0.15 (−0.48, 0.18; 0.372)		−0.07 (−0.41, 0.27; 0.674)		0.00 (−0.35, 0.35; 0.989)		−0.09 (−0.30, 0.12; 0.422)
	EIM vs. UC			−0.06 (−0.40, 0.28; 0.715)		0.04 (−0.30, 0.39; 0.803)		0.10 (−0.26, 0.45; 0.585)		0.11 (−0.11, 0.32; 0.332)
	TCQ vs. EIM			−0.09 (−0.38, 0.20; 0.556)		−0.12 (−0.41, 0.17; 0.429)		−0.10 (−0.39, 0.20; 0.516)		−0.11 (−0.41, 0.18; 0.455)
psqi_disturbances	TCQ	1.44 (0.09)	1.37 (0.09)	−0.08 (−0.26, 0.11; 0.428)	1.44 (0.09)	−0.01 (−0.19, 0.18; 0.951)	1.44 (0.09)	−0.01 (−0.19, 0.18; 0.951)	1.49 (0.10)	0.04 (−0.16, 0.24; 0.679)
	EIM	1.51 (0.09)	1.32 (0.10)	−0.19 (−0.39, 0.01; 0.066)	1.40 (0.10)	−0.11 (−0.31, 0.09; 0.279)	1.36 (0.10)	−0.15 (−0.35, 0.05; 0.151)	1.46 (0.10)	−0.05 (−0.26, 0.15; 0.622)
	UC	1.52 (0.12)	1.40 (0.13)	−0.12 (−0.37, 0.14; 0.366)	1.41 (0.13)	−0.11 (−0.37, 0.15; 0.414)	1.33 (0.13)	−0.19 (−0.46, 0.08; 0.172)	1.57 (0.14)	0.05 (−0.23, 0.34; 0.709)
	TCQ vs. UC			0.04 (−0.27, 0.35; 0.799)		0.10 (−0.22, 0.42; 0.528)		0.18 (−0.15, 0.51; 0.278)		0.04 (−0.16, 0.24; 0.679)
	EIM vs. UC			−0.07 (−0.40, 0.25; 0.652)		−0.00 (−0.33, 0.33; 0.989)		0.04 (−0.30, 0.38; 0.811)		−0.05 (−0.26, 0.15; 0.622)
	TCQ vs. EIM			0.11 (−0.16, 0.39; 0.413)		0.10 (−0.17, 0.38; 0.451)		0.14 (−0.13, 0.42; 0.314)		0.05 (−0.23, 0.34; 0.709)
psqi_duration	TCQ	0.89 (0.13)	0.68 (0.14)	−0.21 (−0.43, 0.00; 0.055)	0.67 (0.14)	−0.22 (−0.44, −0.01; 0.044)	0.70 (0.14)	−0.18 (−0.40, 0.04; 0.100)	0.71 (0.14)	−0.18 (−0.41, 0.05; 0.133)
	EIM	0.70 (0.13)	0.51 (0.15)	−0.19 (−0.43, 0.05; 0.114)	0.66 (0.15)	−0.04 (−0.28, 0.20; 0.753)	0.54 (0.14)	−0.15 (−0.39, 0.08; 0.201)	0.79 (0.15)	0.10 (−0.15, 0.34; 0.436)
	UC	0.64 (0.18)	0.43 (0.19)	−0.21 (−0.51, 0.09; 0.161)	0.47 (0.19)	−0.17 (−0.47, 0.13; 0.268)	0.41 (0.19)	−0.23 (−0.55, 0.08; 0.151)	0.53 (0.20)	−0.11 (−0.44, 0.22; 0.517)
	TCQ vs. UC			0.00 (−0.37, 0.37; 0.998)		−0.05 (−0.42, 0.32; 0.777)		0.05 (−0.34, 0.43; 0.810)		−0.18 (−0.41, 0.05; 0.133)
	EIM vs. UC			0.02 (−0.36, 0.40; 0.909)		0.13 (−0.25, 0.51; 0.502)		0.08 (−0.32, 0.47; 0.696)		0.10 (−0.15, 0.34; 0.436)
	TCQ vs. EIM			−0.02 (−0.34, 0.30; 0.894)		−0.18 (−0.50, 0.14; 0.261)		−0.03 (−0.35, 0.29; 0.849)		−0.11 (−0.44, 0.22; 0.517)
psqi_efficiency	TCQ	0.58 (0.14)	0.56 (0.14)	−0.01 (−0.30, 0.27; 0.925)	0.66 (0.14)	0.08 (−0.20, 0.36; 0.566)	0.56 (0.14)	−0.02 (−0.30, 0.27; 0.915)	0.61 (0.15)	0.03 (−0.27, 0.33; 0.840)
	EIM	0.93 (0.14)	0.55 (0.16)	−0.38 (−0.69, −0.08; 0.014)	0.69 (0.16)	−0.24 (−0.55, 0.06; 0.116)	0.48 (0.16)	−0.46 (−0.76, −0.15; 0.004)	0.86 (0.16)	−0.07 (−0.38, 0.24; 0.650)
	UC	0.44 (0.18)	0.38 (0.20)	−0.06 (−0.44, 0.32; 0.760)	0.49 (0.20)	0.05 (−0.33, 0.44; 0.791)	0.31 (0.21)	−0.13 (−0.53, 0.28; 0.537)	0.37 (0.22)	−0.07 (−0.50, 0.35; 0.735)
	TCQ vs. UC			0.05 (−0.43, 0.52; 0.849)		0.03 (−0.45, 0.50; 0.903)		0.11 (−0.38, 0.61; 0.656)		0.03 (−0.27, 0.33; 0.840)
	EIM vs. UC			−0.32 (−0.82, 0.17; 0.195)		−0.30 (−0.79, 0.19; 0.236)		−0.33 (−0.84, 0.18; 0.209)		−0.07 (−0.38, 0.24; 0.650)
	TCQ vs. EIM			0.37 (−0.05, 0.79; 0.081)		0.33 (−0.09, 0.74; 0.122)		0.44 (0.02, 0.86; 0.040)		−0.07 (−0.50, 0.35; 0.735)
psqi_latency	TCQ	1.13 (0.12)	0.93 (0.13)	−0.20 (−0.44, 0.04; 0.104)	0.90 (0.13)	−0.23 (−0.47, 0.01; 0.060)	0.95 (0.13)	−0.18 (−0.42, 0.07; 0.150)	1.12 (0.14)	−0.02 (−0.27, 0.24; 0.904)
	EIM	1.00 (0.13)	0.62 (0.14)	−0.38 (−0.64, −0.11; 0.005)	0.74 (0.14)	−0.25 (−0.52, 0.01; 0.061)	0.84 (0.14)	−0.16 (−0.42, 0.10; 0.230)	0.87 (0.14)	−0.12 (−0.39, 0.14; 0.365)
	UC	0.76 (0.17)	1.02 (0.18)	0.26 (−0.07, 0.59; 0.119)	0.74 (0.18)	−0.02 (−0.36, 0.32; 0.906)	0.82 (0.19)	0.06 (−0.30, 0.41; 0.756)	0.86 (0.20)	0.10 (−0.27, 0.47; 0.592)
	TCQ vs. UC			−0.46 (−0.87, −0.05; 0.027)		−0.21 (−0.62, 0.20; 0.320)		−0.23 (−0.66, 0.19; 0.282)		−0.02 (−0.27, 0.24; 0.904)
	EIM vs. UC			−0.64 (−1.06, −0.22; 0.003)		−0.23 (−0.66, 0.20; 0.286)		−0.22 (−0.65, 0.22; 0.333)		−0.12 (−0.39, 0.14; 0.365)
	TCQ vs. EIM			0.18 (−0.18, 0.54; 0.327)		0.02 (−0.33, 0.38; 0.897)		−0.02 (−0.38, 0.34; 0.919)		0.10 (−0.27, 0.47; 0.592)
psqi_medication	TCQ	0.49 (0.16)	0.50 (0.16)	0.00 (−0.24, 0.25; 0.974)	0.56 (0.16)	0.07 (−0.17, 0.31; 0.587)	0.40 (0.17)	−0.09 (−0.34, 0.16; 0.472)	0.48 (0.17)	−0.01 (−0.27, 0.25; 0.948)
	EIM	0.60 (0.16)	0.57 (0.17)	−0.03 (−0.30, 0.23; 0.811)	0.53 (0.17)	−0.08 (−0.34, 0.18; 0.565)	0.51 (0.17)	−0.09 (−0.35, 0.17; 0.495)	0.73 (0.18)	0.13 (−0.14, 0.40; 0.345)
	UC	0.76 (0.21)	0.61 (0.22)	−0.15 (−0.48, 0.17; 0.352)	0.67 (0.23)	−0.09 (−0.43, 0.25; 0.591)	0.64 (0.23)	−0.12 (−0.47, 0.23; 0.498)	0.84 (0.24)	0.08 (−0.28, 0.45; 0.655)
	TCQ vs. UC			0.16 (−0.25, 0.57; 0.444)		0.16 (−0.26, 0.57; 0.452)		0.03 (−0.40, 0.46; 0.887)		−0.01 (−0.27, 0.25; 0.948)
	EIM vs. UC			0.12 (−0.30, 0.54; 0.566)		0.02 (−0.41, 0.44; 0.942)		0.03 (−0.41, 0.47; 0.892)		0.13 (−0.14, 0.40; 0.345)
	TCQ vs. EIM			0.04 (−0.32, 0.40; 0.843)		0.14 (−0.21, 0.50; 0.429)		0.00 (−0.36, 0.36; 0.997)		0.08 (−0.28, 0.45; 0.655)
psqi_quality	TCQ	1.16 (0.10)	1.09 (0.10)	−0.07 (−0.23, 0.10; 0.427)	0.99 (0.10)	−0.16 (−0.32, −0.00; 0.049)	1.15 (0.10)	−0.00 (−0.17, 0.16; 0.995)	1.16 (0.10)	0.00 (−0.17, 0.18; 0.973)
	EIM	1.21 (0.10)	1.14 (0.11)	−0.07 (−0.24, 0.11; 0.471)	1.09 (0.11)	−0.12 (−0.30, 0.06; 0.177)	1.06 (0.11)	−0.15 (−0.33, 0.03; 0.100)	1.17 (0.11)	−0.04 (−0.22, 0.14; 0.665)
	UC	1.12 (0.13)	1.00 (0.14)	−0.12 (−0.34, 0.10; 0.287)	0.96 (0.14)	−0.16 (−0.39, 0.07; 0.167)	0.91 (0.14)	−0.21 (−0.45, 0.03; 0.085)	0.93 (0.15)	−0.19 (−0.44, 0.06; 0.130)
	TCQ vs. UC			0.05 (−0.22, 0.33; 0.701)		−0.00 (−0.28, 0.28; 0.989)		0.21 (−0.08, 0.50; 0.157)		0.00 (−0.17, 0.18; 0.973)
	EIM vs. UC			0.05 (−0.23, 0.34; 0.707)		0.04 (−0.25, 0.33; 0.790)		0.06 (−0.24, 0.36; 0.687)		−0.04 (−0.22, 0.14; 0.665)
	TCQ vs. EIM			−0.00 (−0.24, 0.24; 0.996)		−0.04 (−0.28, 0.20; 0.737)		0.15 (−0.09, 0.39; 0.230)		−0.19 (−0.44, 0.06; 0.130)

## Data Availability

Deidentified data from this study are not available in a public archive but can be made available by emailing the corresponding author.
