# Supplementary material for: Effects of Tai Chi Qigong on sleep quality in older male cancer survivors: A randomized controlled clinical trial
Source: Brain Med. Author manuscript; Available in PMC 2026 Sep 29. (PMC13618391; doi:10.61373/bm026a.0063)
Supplement: Supplementary file 1 [file NIHMS2209801-supplement-Supplementary_Tables.pdf]

Supplemental Table 1. Changes in Sleep from Baseline in All Participants: Linear Mixed Model Analysis for Within and Between-Arm Comparison Based on Multiple Imputation (N=113)

		Baseline	6-week mid-intervention		1-week post-intervention		3-month post-intervention		12-month post-intervention	
		**Mean (SE)**	**Mean (SE)**	**Change from baseline Mean Difference (95% CI; p-value)**	**Mean (SE)**	**Change from baseline Mean Difference (95% CI; p-value)**	**Mean (SE)**	**Change from baseline Mean Difference (95% CI; p-value)**	**Mean (SE)**	**Change from baseline Mean Difference (95% CI; p-value)**
PSQI	TCQ	6.43 (0.49)	5.65 (0.48)	-0.79 (-1.57, -0.01; 0.048)	5.74 (0.53)	-0.70 (-1.54, 0.14; 0.104)	5.82 (0.51)	-0.61 (-1.51, 0.29; 0.185)	6.40 (0.57)	-0.03 (-0.94, 0.87; 0.940)
	EIM	6.69 (0.59)	5.52 (0.55)	-1.17 (-2.03, -0.32; 0.007)	5.76 (0.53)	-0.93 (-1.72, -0.15; 0.020)	5.43 (0.57)	-1.26 (-2.15, -0.36; 0.006)	6.58 (0.59)	-0.12 (-1.09, 0.86; 0.816)
	UC	6.24 (0.56)	5.89 (0.50)	-0.35 (-1.40, 0.69; 0.510)	5.68 (0.55)	-0.56 (-1.61, 0.49; 0.297)	5.47 (0.58)	-0.77 (-1.95, 0.41; 0.201)	6.17 (0.65)	-0.07 (-1.27, 1.13; 0.907)
	TCQ vs. UC			-0.44 (-1.72, 0.85; 0.507)		-0.14 (-1.48, 1.21; 0.842)		0.16 (-1.30, 1.61; 0.831)		-0.03 (-0.94, 0.87; 0.940)
	EIM vs. UC			-0.82 (-2.18, 0.54; 0.235)		-0.37 (-1.69, 0.95; 0.578)		-0.49 (-1.94, 0.96; 0.505)		-0.12 (-1.09, 0.86; 0.816)
	TCQ vs. EIM			0.39 (-0.75, 1.52; 0.504)		0.24 (-0.91, 1.38; 0.684)		0.65 (-0.52, 1.82; 0.276)		-0.07 (-1.27, 1.13; 0.907)
psqi_daytime	TCQ	0.73 (0.08)	0.61 (0.10)	-0.11 (-0.30, 0.08; 0.246)	0.59 (0.10)	-0.14 (-0.34, 0.07; 0.186)	0.64 (0.13)	-0.08 (-0.31, 0.14; 0.481)	0.73 (0.12)	0.01 (-0.22, 0.24; 0.947)
	EIM	0.74 (0.11)	0.71 (0.13)	-0.03 (-0.28, 0.22; 0.811)	0.69 (0.13)	-0.06 (-0.26, 0.15; 0.598)	0.70 (0.14)	-0.04 (-0.29, 0.21; 0.746)	0.84 (0.14)	0.09 (-0.12, 0.31; 0.393)
	UC	1.00 (0.10)	0.98 (0.14)	-0.02 (-0.34, 0.30; 0.909)	0.88 (0.13)	-0.12 (-0.40, 0.16; 0.405)	0.91 (0.16)	-0.09 (-0.43, 0.25; 0.597)	0.94 (0.14)	-0.06 (-0.36, 0.24; 0.688)
	TCQ vs. UC			-0.09 (-0.47, 0.28; 0.623)		-0.02 (-0.38, 0.34; 0.912)		0.01 (-0.40, 0.42; 0.963)		0.01 (-0.22, 0.24; 0.947)
	EIM vs. UC			-0.01 (-0.41, 0.39; 0.954)		0.06 (-0.30, 0.42; 0.733)		0.05 (-0.36, 0.46; 0.808)		0.09 (-0.12, 0.31; 0.393)
	TCQ vs. EIM			-0.08 (-0.38, 0.22; 0.593)		-0.08 (-0.37, 0.20; 0.567)		-0.04 (-0.36, 0.28; 0.806)		-0.06 (-0.36, 0.24; 0.688)
psqi_disturbances	TCQ	1.44 (0.09)	1.36 (0.10)	-0.08 (-0.29, 0.13; 0.458)	1.41 (0.09)	-0.03 (-0.25, 0.18; 0.763)	1.42 (0.10)	-0.02 (-0.24, 0.19; 0.821)	1.51 (0.10)	0.07 (-0.16, 0.30; 0.551)
	EIM	1.51 (0.09)	1.36 (0.12)	-0.15 (-0.39, 0.09; 0.208)	1.40 (0.10)	-0.11 (-0.32, 0.10; 0.313)	1.34 (0.11)	-0.18 (-0.43, 0.07; 0.169)	1.48 (0.11)	-0.03 (-0.28, 0.22; 0.824)
	UC	1.52 (0.11)	1.41 (0.14)	-0.11 (-0.36, 0.14; 0.389)	1.41 (0.13)	-0.11 (-0.34, 0.12; 0.355)	1.32 (0.12)	-0.20 (-0.45, 0.04; 0.108)	1.52 (0.15)	0.00 (-0.27, 0.28; 0.973)
	TCQ vs. UC			0.03 (-0.29, 0.35; 0.859)		0.08 (-0.25, 0.40; 0.641)		0.18 (-0.14, 0.50; 0.274)		0.07 (-0.16, 0.30; 0.551)
	EIM vs. UC			-0.05 (-0.39, 0.30; 0.797)		0.00 (-0.32, 0.32; 0.987)		0.03 (-0.32, 0.37; 0.873)		-0.03 (-0.28, 0.22; 0.824)
	TCQ vs. EIM			0.07 (-0.24, 0.39; 0.646)		0.07 (-0.23, 0.38; 0.632)		0.15 (-0.17, 0.47; 0.355)		0.00 (-0.27, 0.28; 0.973)
psqi_duration	TCQ	0.89 (0.15)	0.62 (0.13)	-0.27 (-0.49, -0.06; 0.013)	0.63 (0.13)	-0.26 (-0.51, -0.01; 0.038)	0.71 (0.15)	-0.18 (-0.39, 0.04; 0.113)	0.71 (0.17)	-0.18 (-0.49, 0.13; 0.266)
	EIM	0.70 (0.13)	0.49 (0.12)	-0.20 (-0.41, 0.00; 0.054)	0.60 (0.14)	-0.10 (-0.36, 0.16; 0.458)	0.55 (0.13)	-0.14 (-0.36, 0.07; 0.192)	0.76 (0.17)	0.06 (-0.28, 0.40; 0.725)
	UC	0.64 (0.18)	0.43 (0.12)	-0.21 (-0.47, 0.04; 0.093)	0.48 (0.16)	-0.16 (-0.43, 0.10; 0.229)	0.45 (0.15)	-0.19 (-0.44, 0.06; 0.136)	0.62 (0.22)	-0.02 (-0.37, 0.33; 0.908)
	TCQ vs. UC			-0.06 (-0.39, 0.28; 0.729)		-0.10 (-0.45, 0.26; 0.588)		0.02 (-0.32, 0.35; 0.924)		-0.18 (-0.49, 0.13; 0.266)
	EIM vs. UC			0.01 (-0.31, 0.33; 0.952)		0.06 (-0.31, 0.43; 0.732)		0.05 (-0.28, 0.37; 0.780)		0.06 (-0.28, 0.40; 0.725)
	TCQ vs. EIM			-0.07 (-0.36, 0.23; 0.648)		-0.16 (-0.52, 0.20; 0.376)		-0.03 (-0.34, 0.28; 0.847)		-0.02 (-0.37, 0.33; 0.908)
psqi_efficiency	TCQ	0.58 (0.14)	0.55 (0.14)	-0.03 (-0.36, 0.30; 0.862)	0.62 (0.15)	0.04 (-0.29, 0.38; 0.796)	0.53 (0.12)	-0.05 (-0.37, 0.28; 0.777)	0.65 (0.19)	0.08 (-0.35, 0.50; 0.729)
	EIM	0.93 (0.17)	0.55 (0.17)	-0.38 (-0.70, -0.05; 0.023)	0.64 (0.17)	-0.29 (-0.60, 0.03; 0.074)	0.45 (0.16)	-0.48 (-0.75, -0.21; <.001)	0.76 (0.20)	-0.17 (-0.59, 0.25; 0.427)
	UC	0.44 (0.15)	0.42 (0.15)	-0.02 (-0.31, 0.26; 0.865)	0.51 (0.19)	0.07 (-0.27, 0.42; 0.678)	0.35 (0.15)	-0.09 (-0.42, 0.23; 0.577)	0.45 (0.24)	0.01 (-0.49, 0.51; 0.965)
	TCQ vs. UC			-0.00 (-0.44, 0.43; 0.984)		-0.03 (-0.50, 0.45; 0.905)		0.05 (-0.42, 0.51; 0.845)		0.08 (-0.35, 0.50; 0.729)
	EIM vs. UC			-0.35 (-0.77, 0.07; 0.100)		-0.36 (-0.81, 0.08; 0.110)		-0.39 (-0.82, 0.05; 0.081)		-0.17 (-0.59, 0.25; 0.427)
	TCQ vs. EIM			0.35 (-0.11, 0.80; 0.134)		0.33 (-0.12, 0.79; 0.151)		0.43 (0.01, 0.85; 0.043)		0.01 (-0.49, 0.51; 0.965)
psqi_latency	TCQ	1.13 (0.13)	0.93 (0.13)	-0.21 (-0.42, 0.01; 0.059)	0.92 (0.14)	-0.22 (-0.49, 0.06; 0.121)	0.98 (0.14)	-0.16 (-0.45, 0.13; 0.289)	1.10 (0.15)	-0.03 (-0.28, 0.21; 0.781)
	EIM	0.99 (0.14)	0.70 (0.13)	-0.29 (-0.53, -0.05; 0.017)	0.75 (0.14)	-0.25 (-0.52, 0.02; 0.075)	0.82 (0.15)	-0.18 (-0.44, 0.09; 0.185)	0.87 (0.16)	-0.13 (-0.42, 0.17; 0.401)
	UC	0.76 (0.15)	0.99 (0.18)	0.23 (-0.14, 0.60; 0.220)	0.76 (0.16)	0.00 (-0.39, 0.39; 0.990)	0.82 (0.17)	0.06 (-0.36, 0.48; 0.789)	0.89 (0.19)	0.13 (-0.34, 0.60; 0.590)
	TCQ vs. UC			-0.44 (-0.87, -0.01; 0.047)		-0.22 (-0.69, 0.26; 0.365)		-0.21 (-0.73, 0.30; 0.412)		-0.03 (-0.28, 0.21; 0.781)
	EIM vs. UC			-0.52 (-0.97, -0.07; 0.023)		-0.25 (-0.72, 0.22; 0.299)		-0.24 (-0.73, 0.26; 0.347)		-0.13 (-0.42, 0.17; 0.401)
	TCQ vs. EIM			0.08 (-0.23, 0.39; 0.601)		0.03 (-0.35, 0.41; 0.881)		0.02 (-0.36, 0.40; 0.914)		0.13 (-0.34, 0.60; 0.590)

psqi_medication	TCQ	0.51 (0.16)	0.48 (0.16)	-0.03 (-0.29, 0.23; 0.838)	0.57 (0.16)	0.06 (-0.22, 0.34; 0.689)	0.40 (0.15)	-0.11 (-0.44, 0.22; 0.512)	0.53 (0.18)	0.02 (-0.30, 0.34; 0.909)
	EIM	0.60 (0.17)	0.57 (0.18)	-0.03 (-0.21, 0.15; 0.720)	0.60 (0.19)	-0.01 (-0.28, 0.26; 0.952)	0.51 (0.19)	-0.10 (-0.38, 0.19; 0.512)	0.71 (0.19)	0.11 (-0.15, 0.37; 0.413)
	UC	0.76 (0.22)	0.64 (0.22)	-0.12 (-0.30, 0.07; 0.214)	0.70 (0.23)	-0.06 (-0.44, 0.32; 0.742)	0.67 (0.24)	-0.09 (-0.52, 0.33; 0.659)	0.76 (0.28)	-0.00 (-0.40, 0.40; 0.997)
	TCQ vs. UC			0.09 (-0.23, 0.41; 0.575)		0.12 (-0.36, 0.60; 0.618)		-0.02 (-0.55, 0.52; 0.954)		0.02 (-0.30, 0.34; 0.909)
	EIM vs. UC			0.09 (-0.17, 0.34; 0.517)		0.06 (-0.42, 0.53; 0.821)		-0.00 (-0.51, 0.51; 0.999)		0.11 (-0.15, 0.37; 0.413)
	TCQ vs. EIM			0.01 (-0.30, 0.32; 0.971)		0.07 (-0.32, 0.45; 0.736)		-0.02 (-0.44, 0.41; 0.944)		-0.00 (-0.40, 0.40; 0.997)
psqi_quality	TCQ	1.16 (0.11)	1.10 (0.10)	-0.06 (-0.22, 0.10; 0.473)	1.01 (0.11)	-0.15 (-0.32, 0.02; 0.087)	1.14 (0.11)	-0.01 (-0.19, 0.17; 0.874)	1.16 (0.11)	0.00 (-0.18, 0.19; 0.974)
	EIM	1.21 (0.09)	1.13 (0.12)	-0.08 (-0.26, 0.10; 0.367)	1.08 (0.11)	-0.13 (-0.30, 0.04; 0.135)	1.06 (0.11)	-0.15 (-0.33, 0.04; 0.114)	1.15 (0.11)	-0.06 (-0.27, 0.15; 0.597)
	UC	1.12 (0.12)	1.02 (0.11)	-0.10 (-0.33, 0.13; 0.407)	0.94 (0.13)	-0.18 (-0.43, 0.07; 0.161)	0.97 (0.12)	-0.15 (-0.40, 0.09; 0.224)	0.99 (0.12)	-0.13 (-0.35, 0.08; 0.221)
	TCQ vs. UC			0.04 (-0.24, 0.32; 0.776)		0.03 (-0.27, 0.34; 0.835)		0.14 (-0.16, 0.44; 0.372)		0.00 (-0.18, 0.19; 0.974)
	EIM vs. UC			0.02 (-0.28, 0.31; 0.918)		0.05 (-0.26, 0.37; 0.749)		0.01 (-0.29, 0.31; 0.968)		-0.06 (-0.27, 0.15; 0.597)
	TCQ vs. EIM			0.03 (-0.22, 0.27; 0.838)		-0.02 (-0.26, 0.22; 0.878)		0.13 (-0.12, 0.38; 0.297)		-0.13 (-0.35, 0.08; 0.221)

Supplemental Table 2. Changes in Sleep from Baseline in Participants with Prostate Cancer: Linear Mixed Model Analysis for Within and Between-Arm Comparison (N=98)

		Baseline	6-week mid-intervention		1-week post-intervention		3-month post-intervention		12-month post-intervention	
		Mean (SE)	Mean (SE)	Change from baseline MD (95% CI; p-value)	Mean (SE)	Change from baseline MD (95% CI; p-value)	Mean (SE)	Change from baseline MD (95% CI; p-value)	Mean (SE)	Change from baseline MD (95% CI; p-value)
PSQI	TCQ	6.08 (0.51)	5.35 (0.53)	-0.73 (-1.53, 0.06; 0.071)	5.48 (0.53)	-0.60 (-1.38, 0.18; 0.128)	5.76 (0.53)	-0.32 (-1.11, 0.46; 0.420)	5.81 (0.55)	-0.27 (-1.10, 0.56; 0.519)
	EIM	6.41 (0.54)	5.24 (0.57)	-1.18 (-2.04, -0.32; 0.008)	5.60 (0.57)	-0.82 (-1.68, 0.04; 0.062)	5.37 (0.56)	-1.04 (-1.88, -0.20; 0.015)	6.76 (0.57)	0.35 (-0.52, 1.22; 0.430)
	UC	6.24 (0.63)	5.91 (0.66)	-0.33 (-1.32, 0.65; 0.507)	5.60 (0.67)	-0.64 (-1.66, 0.38; 0.221)	5.30 (0.69)	-0.94 (-2.00, 0.12; 0.083)	5.98 (0.71)	-0.26 (-1.37, 0.85; 0.650)
	TCQ vs. UC			-0.40 (-1.67, 0.87; 0.537)		0.03 (-1.25, 1.32; 0.960)		0.62 (-0.71, 1.94; 0.360)		-0.27 (-1.10, 0.56; 0.519)
	EIM vs. UC			-0.84 (-2.15, 0.47; 0.207)		-0.18 (-1.52, 1.15; 0.791)		-0.10 (-1.46, 1.25; 0.884)		0.35 (-0.52, 1.22; 0.430)
	TCQ vs. EIM			0.44 (-0.73, 1.62; 0.456)		0.21 (-0.95, 1.37; 0.718)		0.72 (-0.43, 1.87; 0.222)		-0.26 (-1.37, 0.85; 0.650)
psqi_daytime	TCQ	0.74 (0.11)	0.59 (0.11)	-0.15 (-0.35, 0.06; 0.158)	0.59 (0.11)	-0.15 (-0.35, 0.05; 0.151)	0.65 (0.11)	-0.08 (-0.29, 0.12; 0.424)	0.57 (0.12)	-0.16 (-0.38, 0.05; 0.134)
	EIM	0.74 (0.11)	0.69 (0.12)	-0.05 (-0.28, 0.17; 0.628)	0.66 (0.12)	-0.08 (-0.30, 0.14; 0.464)	0.70 (0.12)	-0.04 (-0.26, 0.17; 0.699)	0.88 (0.12)	0.14 (-0.09, 0.36; 0.237)
	UC	1.00 (0.13)	1.03 (0.14)	0.03 (-0.23, 0.28; 0.835)	0.91 (0.14)	-0.09 (-0.36, 0.17; 0.494)	0.90 (0.15)	-0.10 (-0.38, 0.17; 0.455)	0.89 (0.15)	-0.11 (-0.40, 0.18; 0.445)
	TCQ vs. UC			-0.17 (-0.50, 0.15; 0.295)		-0.06 (-0.39, 0.28; 0.740)		0.02 (-0.32, 0.36; 0.902)		-0.16 (-0.38, 0.05; 0.134)
	EIM vs. UC			-0.08 (-0.42, 0.26; 0.635)		0.01 (-0.33, 0.35; 0.953)		0.06 (-0.29, 0.41; 0.729)		0.14 (-0.09, 0.36; 0.237)
	TCQ vs. EIM			-0.09 (-0.40, 0.21; 0.545)		-0.07 (-0.36, 0.23; 0.663)		-0.04 (-0.34, 0.26; 0.791)		-0.11 (-0.40, 0.18; 0.445)
psqi_disturbances	TCQ	1.45 (0.09)	1.35 (0.10)	-0.09 (-0.30, 0.11; 0.359)	1.37 (0.10)	-0.08 (-0.28, 0.12; 0.444)	1.42 (0.10)	-0.03 (-0.23, 0.17; 0.770)	1.44 (0.11)	-0.01 (-0.22, 0.20; 0.914)
	EIM	1.46 (0.10)	1.32 (0.11)	-0.14 (-0.36, 0.08; 0.202)	1.44 (0.11)	-0.02 (-0.24, 0.20; 0.854)	1.42 (0.11)	-0.04 (-0.25, 0.18; 0.731)	1.51 (0.11)	0.05 (-0.17, 0.27; 0.656)
	UC	1.52 (0.12)	1.40 (0.12)	-0.12 (-0.37, 0.14; 0.365)	1.41 (0.13)	-0.11 (-0.37, 0.15; 0.413)	1.33 (0.13)	-0.19 (-0.46, 0.08; 0.172)	1.57 (0.14)	0.05 (-0.23, 0.34; 0.709)
	TCQ vs. UC			0.02 (-0.30, 0.34; 0.897)		0.03 (-0.30, 0.36; 0.854)		0.16 (-0.18, 0.49; 0.356)		-0.01 (-0.22, 0.20; 0.914)
	EIM vs. UC			-0.03 (-0.36, 0.31; 0.877)		0.09 (-0.25, 0.43; 0.609)		0.15 (-0.19, 0.49; 0.390)		0.05 (-0.17, 0.27; 0.656)
	TCQ vs. EIM			0.05 (-0.25, 0.35; 0.754)		-0.06 (-0.35, 0.24; 0.701)		0.01 (-0.29, 0.30; 0.960)		0.05 (-0.23, 0.34; 0.709)
psqi_duration	TCQ	0.84 (0.14)	0.62 (0.15)	-0.23 (-0.47, 0.02; 0.066)	0.63 (0.14)	-0.21 (-0.45, 0.02; 0.079)	0.68 (0.14)	-0.16 (-0.40, 0.08; 0.178)	0.65 (0.15)	-0.19 (-0.44, 0.06; 0.142)
	EIM	0.63 (0.15)	0.45 (0.16)	-0.18 (-0.44, 0.08; 0.182)	0.55 (0.16)	-0.08 (-0.34, 0.19; 0.566)	0.44 (0.15)	-0.19 (-0.44, 0.07; 0.151)	0.75 (0.16)	0.12 (-0.15, 0.38; 0.387)
	UC	0.64 (0.17)	0.42 (0.18)	-0.22 (-0.52, 0.09; 0.166)	0.47 (0.18)	-0.17 (-0.48, 0.13; 0.272)	0.41 (0.19)	-0.23 (-0.56, 0.09; 0.156)	0.53 (0.20)	-0.11 (-0.45, 0.22; 0.511)
	TCQ vs. UC			-0.01 (-0.40, 0.38; 0.955)		-0.04 (-0.43, 0.34; 0.831)		0.07 (-0.33, 0.47; 0.736)		-0.19 (-0.44, 0.06; 0.142)
	EIM vs. UC			0.04 (-0.36, 0.44; 0.855)		0.09 (-0.31, 0.50; 0.644)		0.05 (-0.37, 0.46; 0.824)		0.12 (-0.15, 0.38; 0.387)
	TCQ vs. EIM			-0.05 (-0.41, 0.31; 0.788)		-0.14 (-0.49, 0.22; 0.447)		0.02 (-0.33, 0.37; 0.901)		-0.11 (-0.45, 0.22; 0.511)
psqi_efficiency	TCQ	0.45 (0.14)	0.44 (0.15)	-0.01 (-0.31, 0.29; 0.948)	0.61 (0.15)	0.16 (-0.13, 0.45; 0.278)	0.53 (0.15)	0.08 (-0.21, 0.37; 0.577)	0.52 (0.16)	0.07 (-0.24, 0.38; 0.656)
	EIM	0.83 (0.15)	0.49 (0.16)	-0.34 (-0.65, -0.02; 0.038)	0.62 (0.16)	-0.21 (-0.53, 0.10; 0.188)	0.43 (0.16)	-0.39 (-0.71, -0.08; 0.013)	0.89 (0.16)	0.07 (-0.25, 0.39; 0.687)
	UC	0.44 (0.17)	0.38 (0.19)	-0.06 (-0.43, 0.31; 0.748)	0.49 (0.19)	0.05 (-0.32, 0.42; 0.782)	0.31 (0.20)	-0.13 (-0.52, 0.26; 0.520)	0.37 (0.21)	-0.07 (-0.48, 0.33; 0.723)
	TCQ vs. UC			0.05 (-0.42, 0.52; 0.834)		0.11 (-0.36, 0.57; 0.654)		0.21 (-0.28, 0.70; 0.396)		0.07 (-0.24, 0.38; 0.656)
	EIM vs. UC			-0.27 (-0.76, 0.21; 0.266)		-0.26 (-0.75, 0.22; 0.286)		-0.27 (-0.77, 0.23; 0.293)		0.07 (-0.25, 0.39; 0.687)
	TCQ vs. EIM			0.33 (-0.11, 0.76; 0.140)		0.37 (-0.06, 0.80; 0.089)		0.48 (0.05, 0.90; 0.028)		-0.07 (-0.48, 0.33; 0.723)
psqi_latency	TCQ	1.08 (0.13)	0.82 (0.14)	-0.26 (-0.53, 0.01; 0.057)	0.83 (0.14)	-0.25 (-0.51, 0.02; 0.065)	0.89 (0.14)	-0.19 (-0.45, 0.08; 0.164)	1.01 (0.15)	-0.07 (-0.35, 0.21; 0.608)
	EIM	0.94 (0.14)	0.57 (0.15)	-0.37 (-0.66, -0.08; 0.014)	0.73 (0.15)	-0.20 (-0.49, 0.09; 0.175)	0.83 (0.15)	-0.10 (-0.39, 0.18; 0.474)	0.88 (0.15)	-0.05 (-0.35, 0.24; 0.730)
	UC	0.76 (0.16)	1.02 (0.17)	0.26 (-0.07, 0.59; 0.123)	0.74 (0.18)	-0.02 (-0.36, 0.32; 0.911)	0.82 (0.19)	0.06 (-0.30, 0.41; 0.750)	0.86 (0.19)	0.10 (-0.27, 0.47; 0.589)
	TCQ vs. UC			-0.52 (-0.95, -0.09; 0.017)		-0.23 (-0.66, 0.20; 0.301)		-0.25 (-0.69, 0.20; 0.277)		-0.07 (-0.35, 0.21; 0.608)
	EIM vs. UC			-0.63 (-1.07, -0.19; 0.005)		-0.18 (-0.63, 0.27; 0.428)		-0.16 (-0.62, 0.29; 0.487)		-0.05 (-0.35, 0.24; 0.730)
	TCQ vs. EIM			0.11 (-0.29, 0.50; 0.593)		-0.05 (-0.44, 0.35; 0.819)		-0.08 (-0.47, 0.30; 0.670)		0.10 (-0.27, 0.47; 0.589)
psqi_medication	TCQ	0.39 (0.17)	0.45 (0.18)	0.06 (-0.19, 0.31; 0.643)	0.49 (0.17)	0.10 (-0.15, 0.34; 0.425)	0.44 (0.17)	0.04 (-0.21, 0.29; 0.741)	0.47 (0.18)	0.08 (-0.18, 0.34; 0.565)
	EIM	0.66 (0.18)	0.59 (0.19)	-0.06 (-0.33, 0.21; 0.649)	0.55 (0.19)	-0.10 (-0.37, 0.16; 0.443)	0.53 (0.18)	-0.13 (-0.39, 0.13; 0.334)	0.75 (0.19)	0.09 (-0.18, 0.37; 0.504)
	UC	0.76 (0.21)	0.61 (0.22)	-0.15 (-0.47, 0.16; 0.330)	0.67 (0.22)	-0.09 (-0.41, 0.23; 0.574)	0.64 (0.23)	-0.12 (-0.45, 0.21; 0.482)	0.84 (0.23)	0.08 (-0.26, 0.43; 0.635)

	TCQ vs. UC			0.21 (-0.19, 0.61; 0.294)		0.19 (-0.21, 0.60; 0.352)		0.16 (-0.25, 0.58; 0.446)		0.08 (-0.18, 0.34; 0.565)
	EIM vs. UC			0.09 (-0.32, 0.50; 0.664)		-0.01 (-0.43, 0.41; 0.953)		-0.01 (-0.44, 0.42; 0.961)		0.09 (-0.18, 0.37; 0.504)
	TCQ vs. EIM			0.12 (-0.25, 0.49; 0.516)		0.20 (-0.16, 0.57; 0.270)		0.17 (-0.19, 0.53; 0.352)		0.08 (-0.26, 0.43; 0.635)
psqi_quality	TCQ	1.13 (0.10)	1.03 (0.11)	-0.10 (-0.27, 0.07; 0.254)	0.92 (0.11)	-0.21 (-0.38, -0.04; 0.015)	1.10 (0.11)	-0.03 (-0.20, 0.14; 0.756)	1.11 (0.11)	-0.02 (-0.20, 0.16; 0.795)
	EIM	1.17 (0.11)	1.14 (0.11)	-0.03 (-0.22, 0.15; 0.733)	1.04 (0.11)	-0.13 (-0.31, 0.06; 0.174)	1.01 (0.11)	-0.16 (-0.34, 0.02; 0.084)	1.10 (0.12)	-0.07 (-0.26, 0.12; 0.450)
	UC	1.12 (0.13)	1.00 (0.13)	-0.12 (-0.34, 0.10; 0.273)	0.96 (0.14)	-0.16 (-0.38, 0.06; 0.155)	0.91 (0.14)	-0.21 (-0.44, 0.02; 0.076)	0.93 (0.14)	-0.19 (-0.43, 0.05; 0.119)
	TCQ vs. UC			0.02 (-0.26, 0.30; 0.889)		-0.05 (-0.33, 0.23; 0.729)		0.18 (-0.11, 0.47; 0.214)		-0.02 (-0.20, 0.16; 0.795)
	EIM vs. UC			0.09 (-0.20, 0.37; 0.546)		0.03 (-0.26, 0.32; 0.824)		0.05 (-0.25, 0.34; 0.747)		-0.07 (-0.26, 0.12; 0.450)
	TCQ vs. EIM			-0.07 (-0.32, 0.19; 0.600)		-0.08 (-0.33, 0.17; 0.521)		0.13 (-0.12, 0.38; 0.294)		-0.19 (-0.43, 0.05; 0.119)

Supplemental Table 3. Changes in Sleep in Participants who did not drop out between baseline and mid-intervention: Linear Mixed Model Analysis for Within and Between-Arm Comparison (n=98)

		Baseline	6-week mid-intervention		1-week post-intervention		3-month post-intervention		12-month post-intervention	
		**Mean (SE)**	**Mean (SE)**	**Change from baseline MD (95% CI; p-value)**	**Mean (SE)**	**Change from baseline MD (95% CI; p-value)**	**Mean (SE)**	**Change from baseline MD (95% CI; p-value)**	**Mean (SE)**	**Change from baseline MD (95% CI; p-value)**
PSQI	TCQ	6.30 (0.50)	5.64 (0.51)	-0.66 (-1.40, 0.09; 0.084)	5.69 (0.51)	-0.61 (-1.35, 0.13; 0.105)	5.75 (0.51)	-0.55 (-1.30, 0.21; 0.154)	6.11 (0.53)	-0.19 (-0.97, 0.60; 0.645)
	EIM	6.33 (0.54)	5.22 (0.56)	-1.11 (-1.93, -0.29; 0.008)	5.60 (0.56)	-0.73 (-1.55, 0.09; 0.081)	5.38 (0.55)	-0.95 (-1.76, -0.14; 0.022)	6.54 (0.56)	0.21 (-0.62, 1.04; 0.613)
	UC	6.05 (0.69)	5.76 (0.69)	-0.28 (-1.29, 0.73; 0.584)	5.46 (0.71)	-0.58 (-1.63, 0.46; 0.271)	5.16 (0.72)	-0.89 (-1.97, 0.20; 0.109)	5.84 (0.74)	-0.20 (-1.34, 0.93; 0.723)
	TCQ vs. UC			-0.37 (-1.63, 0.88; 0.558)		-0.03 (-1.30, 1.25; 0.968)		0.34 (-0.98, 1.66; 0.612)		-0.19 (-0.97, 0.60; 0.645)
	EIM vs. UC			-0.83 (-2.13, 0.47; 0.209)		-0.14 (-1.47, 1.18; 0.830)		-0.06 (-1.42, 1.29; 0.929)		0.21 (-0.62, 1.04; 0.613)
	TCQ vs. EIM			0.46 (-0.65, 1.56; 0.417)		0.12 (-0.98, 1.22; 0.833)		0.40 (-0.70, 1.51; 0.475)		-0.20 (-1.34, 0.93; 0.723)
psqi_daytime	TCQ	0.73 (0.10)	0.60 (0.10)	-0.12 (-0.32, 0.08; 0.221)	0.56 (0.10)	-0.16 (-0.36, 0.03; 0.106)	0.62 (0.10)	-0.10 (-0.31, 0.10; 0.310)	0.64 (0.11)	-0.08 (-0.30, 0.13; 0.429)
	EIM	0.66 (0.11)	0.66 (0.11)	0.00 (-0.22, 0.22; 0.987)	0.65 (0.11)	-0.01 (-0.22, 0.21; 0.945)	0.69 (0.11)	0.03 (-0.18, 0.25; 0.776)	0.80 (0.11)	0.15 (-0.07, 0.37; 0.191)
	UC	0.95 (0.14)	1.00 (0.14)	0.05 (-0.22, 0.31; 0.733)	0.88 (0.14)	-0.07 (-0.35, 0.21; 0.618)	0.87 (0.15)	-0.09 (-0.38, 0.20; 0.547)	0.86 (0.16)	-0.09 (-0.39, 0.21; 0.540)
	TCQ vs. UC			-0.17 (-0.51, 0.16; 0.315)		-0.09 (-0.43, 0.25; 0.592)		-0.02 (-0.37, 0.34; 0.929)		-0.08 (-0.30, 0.13; 0.429)
	EIM vs. UC			-0.04 (-0.39, 0.30; 0.799)		0.06 (-0.29, 0.41; 0.726)		0.12 (-0.24, 0.48; 0.514)		0.15 (-0.07, 0.37; 0.191)
	TCQ vs. EIM			-0.13 (-0.42, 0.17; 0.401)		-0.16 (-0.45, 0.14; 0.297)		-0.14 (-0.43, 0.16; 0.367)		-0.09 (-0.39, 0.21; 0.540)
psqi_disturbances	TCQ	1.44 (0.09)	1.37 (0.09)	-0.07 (-0.26, 0.11; 0.446)	1.44 (0.09)	-0.00 (-0.19, 0.18; 0.975)	1.44 (0.09)	-0.00 (-0.19, 0.19; 0.974)	1.48 (0.10)	0.04 (-0.15, 0.24; 0.659)
	EIM	1.51 (0.10)	1.32 (0.10)	-0.19 (-0.40, 0.02; 0.069)	1.40 (0.10)	-0.11 (-0.32, 0.09; 0.282)	1.37 (0.10)	-0.15 (-0.35, 0.06; 0.155)	1.46 (0.10)	-0.05 (-0.26, 0.16; 0.619)

	UC	1.55 (0.12)	1.42 (0.12)	-0.13 (-0.38, 0.13; 0.319)	1.42 (0.13)	-0.12 (-0.38, 0.14; 0.366)	1.34 (0.13)	-0.20 (-0.47, 0.07; 0.148)	1.59 (0.14)	0.04 (-0.24, 0.32; 0.779)
	TCQ vs. UC			0.06 (-0.26, 0.37; 0.727)		0.12 (-0.20, 0.44; 0.472)		0.20 (-0.13, 0.53; 0.242)		0.04 (-0.15, 0.24; 0.659)
	EIM vs. UC			-0.06 (-0.39, 0.27; 0.708)		0.01 (-0.32, 0.34; 0.958)		0.05 (-0.29, 0.39; 0.760)		-0.05 (-0.26, 0.16; 0.619)
	TCQ vs. EIM			0.12 (-0.16, 0.40; 0.404)		0.11 (-0.17, 0.39; 0.439)		0.14 (-0.13, 0.42; 0.307)		0.04 (-0.24, 0.32; 0.779)
psqi_duration	TCQ	0.83 (0.13)	0.63 (0.13)	-0.20 (-0.41, 0.02; 0.079)	0.63 (0.13)	-0.20 (-0.42, 0.01; 0.065)	0.66 (0.13)	-0.17 (-0.39, 0.05; 0.134)	0.67 (0.14)	-0.16 (-0.39, 0.07; 0.173)
	EIM	0.57 (0.14)	0.42 (0.15)	-0.15 (-0.39, 0.09; 0.217)	0.57 (0.15)	0.00 (-0.24, 0.24; 0.987)	0.46 (0.15)	-0.11 (-0.35, 0.12; 0.350)	0.71 (0.15)	0.14 (-0.11, 0.38; 0.272)
	UC	0.55 (0.18)	0.36 (0.18)	-0.18 (-0.48, 0.12; 0.231)	0.41 (0.18)	-0.14 (-0.44, 0.16; 0.365)	0.34 (0.19)	-0.20 (-0.52, 0.12; 0.213)	0.46 (0.20)	-0.08 (-0.41, 0.25; 0.633)
	TCQ vs. UC			-0.01 (-0.38, 0.36; 0.948)		-0.07 (-0.44, 0.30; 0.728)		0.03 (-0.35, 0.42; 0.866)		-0.16 (-0.39, 0.07; 0.173)
	EIM vs. UC			0.03 (-0.35, 0.42; 0.870)		0.14 (-0.24, 0.52; 0.472)		0.09 (-0.31, 0.48; 0.662)		0.14 (-0.11, 0.38; 0.272)
	TCQ vs. EIM			-0.04 (-0.37, 0.28; 0.788)		-0.21 (-0.53, 0.12; 0.210)		-0.05 (-0.38, 0.27; 0.739)		-0.08 (-0.41, 0.25; 0.633)
psqi_efficiency	TCQ	0.46 (0.14)	0.51 (0.14)	0.04 (-0.24, 0.33; 0.768)	0.60 (0.14)	0.14 (-0.14, 0.42; 0.333)	0.50 (0.14)	0.04 (-0.24, 0.32; 0.779)	0.55 (0.15)	0.09 (-0.21, 0.38; 0.568)
	EIM	0.77 (0.15)	0.47 (0.15)	-0.31 (-0.61, 0.00; 0.053)	0.61 (0.15)	-0.16 (-0.47, 0.14; 0.294)	0.40 (0.16)	-0.38 (-0.69, -0.06; 0.018)	0.78 (0.16)	0.01 (-0.31, 0.32; 0.967)
	UC	0.41 (0.19)	0.36 (0.19)	-0.04 (-0.43, 0.34; 0.820)	0.48 (0.19)	0.07 (-0.32, 0.45; 0.730)	0.30 (0.20)	-0.11 (-0.52, 0.30; 0.593)	0.35 (0.21)	-0.06 (-0.48, 0.37; 0.790)
	TCQ vs. UC			0.09 (-0.39, 0.57; 0.721)		0.07 (-0.41, 0.55; 0.774)		0.15 (-0.35, 0.65; 0.549)		0.09 (-0.21, 0.38; 0.568)
	EIM vs. UC			-0.26 (-0.76, 0.23; 0.300)		-0.23 (-0.73, 0.26; 0.355)		-0.27 (-0.78, 0.25; 0.311)		0.01 (-0.31, 0.32; 0.967)
	TCQ vs. EIM			0.35 (-0.07, 0.77; 0.103)		0.30 (-0.11, 0.72; 0.154)		0.42 (-0.01, 0.84; 0.053)		-0.06 (-0.48, 0.37; 0.790)

psqi_latency	TCQ	1.12 (0.13)	0.93 (0.13)	-0.20 (-0.44, 0.05; 0.117)	0.90 (0.13)	-0.22 (-0.47, 0.02; 0.069)	0.95 (0.13)	-0.17 (-0.42, 0.07; 0.166)	1.11 (0.14)	-0.01 (-0.27, 0.25; 0.934)
	EIM	0.97 (0.14)	0.60 (0.15)	-0.37 (-0.64, -0.09; 0.008)	0.73 (0.15)	-0.24 (-0.51, 0.03; 0.082)	0.82 (0.14)	-0.15 (-0.42, 0.12; 0.282)	0.86 (0.15)	-0.11 (-0.38, 0.16; 0.429)
	UC	0.77 (0.18)	1.03 (0.18)	0.25 (-0.08, 0.59; 0.132)	0.75 (0.19)	-0.03 (-0.37, 0.32; 0.881)	0.82 (0.19)	0.05 (-0.31, 0.40; 0.784)	0.87 (0.20)	0.09 (-0.28, 0.46; 0.618)
	TCQ vs. UC			-0.45 (-0.86, -0.04; 0.033)		-0.20 (-0.62, 0.22; 0.351)		-0.22 (-0.66, 0.21; 0.310)		-0.01 (-0.27, 0.25; 0.934)
	EIM vs. UC			-0.62 (-1.05, -0.19; 0.005)		-0.21 (-0.65, 0.22; 0.335)		-0.20 (-0.64, 0.25; 0.385)		-0.11 (-0.38, 0.16; 0.429)
	TCQ vs. EIM			0.17 (-0.19, 0.54; 0.358)		0.02 (-0.35, 0.38; 0.935)		-0.03 (-0.39, 0.34; 0.885)		0.09 (-0.28, 0.46; 0.618)
psqi_medication	TCQ	0.54 (0.17)	0.53 (0.17)	-0.01 (-0.25, 0.24; 0.942)	0.59 (0.17)	0.05 (-0.19, 0.30; 0.666)	0.44 (0.17)	-0.10 (-0.35, 0.14; 0.412)	0.52 (0.18)	-0.02 (-0.28, 0.24; 0.867)
	EIM	0.66 (0.18)	0.61 (0.19)	-0.05 (-0.31, 0.22; 0.735)	0.57 (0.19)	-0.09 (-0.36, 0.17; 0.502)	0.55 (0.19)	-0.10 (-0.37, 0.16; 0.437)	0.77 (0.19)	0.11 (-0.16, 0.39; 0.406)
	UC	0.68 (0.23)	0.55 (0.23)	-0.13 (-0.46, 0.20; 0.423)	0.61 (0.24)	-0.07 (-0.41, 0.27; 0.677)	0.58 (0.24)	-0.10 (-0.45, 0.25; 0.577)	0.79 (0.25)	0.10 (-0.27, 0.47; 0.582)
	TCQ vs. UC			0.13 (-0.29, 0.54; 0.548)		0.13 (-0.29, 0.54; 0.555)		-0.00 (-0.44, 0.43; 0.989)		-0.02 (-0.28, 0.24; 0.867)
	EIM vs. UC			0.09 (-0.34, 0.51; 0.682)		-0.02 (-0.45, 0.41; 0.934)		-0.00 (-0.45, 0.44; 0.984)		0.11 (-0.16, 0.39; 0.406)
	TCQ vs. EIM			0.04 (-0.33, 0.40; 0.841)		0.14 (-0.22, 0.50; 0.432)		0.00 (-0.36, 0.36; 0.994)		0.10 (-0.27, 0.47; 0.582)
psqi_quality	TCQ	1.12 (0.10)	1.07 (0.10)	-0.06 (-0.22, 0.11; 0.510)	0.97 (0.10)	-0.15 (-0.31, 0.01; 0.069)	1.13 (0.10)	0.01 (-0.16, 0.18; 0.902)	1.14 (0.11)	0.01 (-0.16, 0.19; 0.875)
	EIM	1.20 (0.11)	1.14 (0.11)	-0.06 (-0.24, 0.12; 0.497)	1.08 (0.11)	-0.12 (-0.30, 0.06; 0.194)	1.05 (0.11)	-0.15 (-0.32, 0.03; 0.111)	1.16 (0.11)	-0.04 (-0.22, 0.15; 0.692)
	UC	1.14 (0.14)	1.01 (0.14)	-0.12 (-0.35, 0.10; 0.271)	0.97 (0.14)	-0.17 (-0.40, 0.06; 0.158)	0.92 (0.15)	-0.21 (-0.45, 0.03; 0.079)	0.94 (0.15)	-0.20 (-0.45, 0.05; 0.122)
	TCQ vs. UC			0.07 (-0.21, 0.35; 0.621)		0.01 (-0.27, 0.30; 0.921)		0.22 (-0.07, 0.52; 0.130)		0.01 (-0.16, 0.19; 0.875)

EIM vs. UC	0.06 (-0.22, 0.35; 0.669)	0.05 (-0.24, 0.34; 0.750)	0.07 (-0.23, 0.37; 0.651)	-0.04 (-0.22, 0.15; 0.692)
TCQ vs. EIM	0.01 (-0.24, 0.25; 0.953)	-0.03 (-0.28, 0.21; 0.788)	0.16 (-0.09, 0.40; 0.211)	-0.20 (-0.45, 0.05; 0.122)

Supplemental Table 4. Changes in Sleep in Participants who did not drop out between baseline and 1-week post-intervention: Linear Mixed Model Analysis for Within and Between-Arm Comparison (n=95)

		Baseline	6-week mid-intervention		1-week post-intervention		3-month post-intervention		12-month post-intervention	
		**Mean (SE)**	**Mean (SE)**	**Change from baseline MD (95% CI; p-value)**	**Mean (SE)**	**Change from baseline MD (95% CI; p-value)**	**Mean (SE)**	**Change from baseline MD (95% CI; p-value)**	**Mean (SE)**	**Change from baseline MD (95% CI; p-value)**
PSQI	TCQ	6.36 (0.51)	5.58 (0.52)	-0.78 (-1.53, -0.03; 0.042)	5.69 (0.51)	-0.67 (-1.41, 0.07; 0.076)	5.75 (0.52)	-0.61 (-1.36, 0.15; 0.114)	6.11 (0.53)	-0.24 (-1.03, 0.54; 0.543)
	EIM	6.34 (0.55)	5.29 (0.57)	-1.05 (-1.88, -0.22; 0.013)	5.63 (0.56)	-0.71 (-1.53, 0.11; 0.091)	5.41 (0.56)	-0.93 (-1.74, -0.11; 0.025)	6.57 (0.57)	0.24 (-0.59, 1.06; 0.575)
	UC	6.24 (0.70)	5.90 (0.70)	-0.33 (-1.34, 0.68; 0.516)	5.60 (0.72)	-0.64 (-1.68, 0.41; 0.230)	5.30 (0.73)	-0.94 (-2.02, 0.14; 0.089)	5.98 (0.75)	-0.26 (-1.39, 0.87; 0.657)
	TCQ vs. UC			-0.44 (-1.70, 0.81; 0.487)		-0.03 (-1.31, 1.24; 0.959)		0.33 (-0.99, 1.65; 0.620)		-0.24 (-1.03, 0.54; 0.543)
	EIM vs. UC			-0.72 (-2.02, 0.59; 0.280)		-0.07 (-1.40, 1.25; 0.917)		0.01 (-1.34, 1.37; 0.985)		0.24 (-0.59, 1.06; 0.575)
	TCQ vs. EIM			0.27 (-0.85, 1.39; 0.632)		0.04 (-1.07, 1.14; 0.947)		0.32 (-0.79, 1.43; 0.569)		-0.26 (-1.39, 0.87; 0.657)
psqi_daytime	TCQ	0.75 (0.10)	0.59 (0.11)	-0.15 (-0.36, 0.05; 0.133)	0.57 (0.10)	-0.18 (-0.38, 0.02; 0.076)	0.63 (0.11)	-0.12 (-0.32, 0.08; 0.242)	0.65 (0.11)	-0.10 (-0.31, 0.11; 0.347)
	EIM	0.65 (0.11)	0.68 (0.12)	0.03 (-0.19, 0.25; 0.759)	0.65 (0.11)	0.01 (-0.21, 0.22; 0.958)	0.69 (0.11)	0.04 (-0.17, 0.26; 0.682)	0.81 (0.12)	0.16 (-0.06, 0.38; 0.152)
	UC	0.95 (0.14)	1.00 (0.14)	0.05 (-0.22, 0.32; 0.727)	0.88 (0.15)	-0.07 (-0.35, 0.21; 0.621)	0.87 (0.15)	-0.09 (-0.37, 0.20; 0.556)	0.86 (0.16)	-0.09 (-0.39, 0.21; 0.546)
	TCQ vs. UC			-0.20 (-0.54, 0.13; 0.238)		-0.11 (-0.45, 0.23; 0.528)		-0.03 (-0.38, 0.32; 0.849)		-0.10 (-0.31, 0.11; 0.347)
	EIM vs. UC			-0.01 (-0.36, 0.33; 0.940)		0.08 (-0.28, 0.43; 0.672)		0.13 (-0.23, 0.49; 0.474)		0.16 (-0.06, 0.38; 0.152)
	TCQ vs. EIM			-0.19 (-0.49, 0.11; 0.214)		-0.18 (-0.48, 0.11; 0.215)		-0.16 (-0.46, 0.13; 0.272)		-0.09 (-0.39, 0.21; 0.546)
psqi_disturbances	TCQ	1.45 (0.09)	1.38 (0.09)	-0.07 (-0.26, 0.12; 0.444)	1.44 (0.09)	-0.01 (-0.19, 0.18; 0.937)	1.44 (0.09)	-0.01 (-0.20, 0.18; 0.937)	1.49 (0.10)	0.04 (-0.16, 0.24; 0.694)
	EIM	1.50 (0.10)	1.33 (0.10)	-0.17 (-0.38, 0.04; 0.121)	1.40 (0.10)	-0.10 (-0.30, 0.11; 0.344)	1.37 (0.10)	-0.13 (-0.34, 0.07; 0.197)	1.46 (0.10)	-0.04 (-0.25, 0.17; 0.710)

	UC	1.57 (0.13)	1.43 (0.13)	-0.14 (-0.40, 0.11; 0.273)	1.44 (0.13)	-0.13 (-0.40, 0.13; 0.316)	1.36 (0.14)	-0.21 (-0.49, 0.06; 0.124)	1.60 (0.14)	0.03 (-0.26, 0.31; 0.855)
	TCQ vs. UC			0.07 (-0.25, 0.39; 0.672)		0.13 (-0.20, 0.45; 0.440)		0.21 (-0.13, 0.54; 0.223)		0.04 (-0.16, 0.24; 0.694)
	EIM vs. UC			-0.02 (-0.35, 0.31; 0.893)		0.04 (-0.30, 0.37; 0.833)		0.08 (-0.26, 0.42; 0.646)		-0.04 (-0.25, 0.17; 0.710)
	TCQ vs. EIM			0.09 (-0.19, 0.37; 0.526)		0.09 (-0.19, 0.37; 0.518)		0.13 (-0.15, 0.41; 0.372)		0.03 (-0.26, 0.31; 0.855)
psqi_duration	TCQ	0.80 (0.13)	0.63 (0.14)	-0.17 (-0.40, 0.05; 0.121)	0.61 (0.13)	-0.19 (-0.41, 0.03; 0.086)	0.65 (0.14)	-0.15 (-0.37, 0.07; 0.172)	0.65 (0.14)	-0.15 (-0.38, 0.09; 0.216)
	EIM	0.56 (0.14)	0.44 (0.15)	-0.12 (-0.37, 0.12; 0.318)	0.57 (0.15)	0.01 (-0.23, 0.26; 0.904)	0.46 (0.15)	-0.10 (-0.34, 0.14; 0.408)	0.71 (0.15)	0.15 (-0.09, 0.39; 0.230)
	UC	0.57 (0.18)	0.38 (0.19)	-0.19 (-0.49, 0.11; 0.211)	0.42 (0.19)	-0.15 (-0.45, 0.15; 0.336)	0.36 (0.19)	-0.21 (-0.53, 0.11; 0.195)	0.48 (0.20)	-0.09 (-0.42, 0.24; 0.598)
	TCQ vs. UC			0.02 (-0.36, 0.39; 0.927)		-0.04 (-0.41, 0.33; 0.822)		0.06 (-0.33, 0.44; 0.774)		-0.15 (-0.38, 0.09; 0.216)
	EIM vs. UC			0.07 (-0.32, 0.46; 0.730)		0.16 (-0.22, 0.55; 0.409)		0.11 (-0.29, 0.51; 0.587)		0.15 (-0.09, 0.39; 0.230)
	TCQ vs. EIM			-0.05 (-0.38, 0.28; 0.762)		-0.20 (-0.53, 0.12; 0.215)		-0.05 (-0.38, 0.27; 0.747)		-0.09 (-0.42, 0.24; 0.598)
psqi_efficiency	TCQ	0.48 (0.14)	0.44 (0.14)	-0.04 (-0.32, 0.24; 0.793)	0.58 (0.14)	0.11 (-0.17, 0.38; 0.438)	0.49 (0.14)	0.01 (-0.27, 0.29; 0.933)	0.53 (0.15)	0.06 (-0.24, 0.35; 0.700)
	EIM	0.79 (0.15)	0.45 (0.16)	-0.35 (-0.66, -0.04; 0.028)	0.61 (0.15)	-0.19 (-0.49, 0.12; 0.231)	0.40 (0.16)	-0.40 (-0.71, -0.09; 0.012)	0.78 (0.16)	-0.01 (-0.32, 0.29; 0.928)
	UC	0.43 (0.19)	0.37 (0.19)	-0.05 (-0.44, 0.33; 0.779)	0.49 (0.19)	0.06 (-0.32, 0.44; 0.767)	0.31 (0.20)	-0.12 (-0.53, 0.28; 0.553)	0.36 (0.21)	-0.07 (-0.49, 0.35; 0.752)
	TCQ vs. UC			0.02 (-0.46, 0.49; 0.945)		0.05 (-0.42, 0.52; 0.831)		0.13 (-0.36, 0.62; 0.593)		0.06 (-0.24, 0.35; 0.700)
	EIM vs. UC			-0.29 (-0.78, 0.20; 0.242)		-0.24 (-0.73, 0.25; 0.328)		-0.28 (-0.78, 0.23; 0.287)		-0.01 (-0.32, 0.29; 0.928)
	TCQ vs. EIM			0.31 (-0.11, 0.73; 0.147)		0.29 (-0.12, 0.71; 0.159)		0.41 (-0.01, 0.83; 0.054)		-0.07 (-0.49, 0.35; 0.752)

psqi_latency	TCQ	1.15 (0.13)	0.92 (0.14)	-0.23 (-0.47, 0.02; 0.073)	0.91 (0.13)	-0.24 (-0.49, -0.00; 0.049)	0.96 (0.14)	-0.19 (-0.44, 0.05; 0.126)	1.12 (0.14)	-0.03 (-0.29, 0.23; 0.822)
	EIM	0.96 (0.14)	0.62 (0.15)	-0.34 (-0.62, -0.07; 0.014)	0.73 (0.15)	-0.23 (-0.50, 0.04; 0.093)	0.83 (0.15)	-0.14 (-0.41, 0.13; 0.309)	0.86 (0.15)	-0.10 (-0.38, 0.17; 0.463)
	UC	0.81 (0.18)	1.05 (0.18)	0.24 (-0.09, 0.57; 0.160)	0.77 (0.19)	-0.04 (-0.38, 0.30; 0.811)	0.84 (0.19)	0.03 (-0.32, 0.39; 0.853)	0.89 (0.20)	0.08 (-0.29, 0.45; 0.680)
	TCQ vs. UC			-0.46 (-0.88, -0.05; 0.028)		-0.20 (-0.62, 0.22; 0.345)		-0.23 (-0.66, 0.21; 0.305)		-0.03 (-0.29, 0.23; 0.822)
	EIM vs. UC			-0.58 (-1.01, -0.15; 0.008)		-0.19 (-0.63, 0.25; 0.391)		-0.17 (-0.62, 0.27; 0.446)		-0.10 (-0.38, 0.17; 0.463)
	TCQ vs. EIM			0.12 (-0.25, 0.49; 0.528)		-0.01 (-0.38, 0.35; 0.952)		-0.05 (-0.42, 0.31; 0.775)		0.08 (-0.29, 0.45; 0.680)
psqi_medication	TCQ	0.55 (0.17)	0.54 (0.17)	-0.01 (-0.26, 0.24; 0.942)	0.60 (0.17)	0.05 (-0.19, 0.30; 0.681)	0.45 (0.17)	-0.11 (-0.36, 0.14; 0.405)	0.53 (0.18)	-0.02 (-0.29, 0.24; 0.855)
	EIM	0.68 (0.19)	0.63 (0.19)	-0.05 (-0.32, 0.23; 0.735)	0.58 (0.19)	-0.09 (-0.36, 0.17; 0.490)	0.57 (0.19)	-0.11 (-0.38, 0.16; 0.425)	0.79 (0.19)	0.11 (-0.16, 0.38; 0.423)
	UC	0.71 (0.24)	0.57 (0.24)	-0.14 (-0.47, 0.19; 0.398)	0.63 (0.24)	-0.08 (-0.42, 0.26; 0.644)	0.61 (0.25)	-0.11 (-0.47, 0.25; 0.549)	0.81 (0.25)	0.10 (-0.28, 0.47; 0.614)
	TCQ vs. UC			0.13 (-0.28, 0.55; 0.527)		0.13 (-0.29, 0.55; 0.539)		0.00 (-0.43, 0.44; 0.989)		-0.02 (-0.29, 0.24; 0.855)
	EIM vs. UC			0.10 (-0.33, 0.53; 0.661)		-0.01 (-0.45, 0.42; 0.952)		0.00 (-0.45, 0.45; 0.999)		0.11 (-0.16, 0.38; 0.423)
	TCQ vs. EIM			0.04 (-0.33, 0.41; 0.841)		0.15 (-0.22, 0.51; 0.432)		0.00 (-0.36, 0.37; 0.989)		0.10 (-0.28, 0.47; 0.614)
psqi_quality	TCQ	1.13 (0.10)	1.07 (0.10)	-0.06 (-0.22, 0.11; 0.505)	0.97 (0.10)	-0.15 (-0.32, 0.01; 0.068)	1.13 (0.10)	0.01 (-0.16, 0.18; 0.914)	1.14 (0.11)	0.01 (-0.16, 0.19; 0.886)
	EIM	1.21 (0.11)	1.14 (0.11)	-0.06 (-0.25, 0.12; 0.493)	1.09 (0.11)	-0.12 (-0.30, 0.06; 0.190)	1.06 (0.11)	-0.15 (-0.33, 0.03; 0.109)	1.17 (0.11)	-0.04 (-0.22, 0.15; 0.679)
	UC	1.19 (0.14)	1.05 (0.14)	-0.14 (-0.37, 0.08; 0.211)	1.01 (0.14)	-0.18 (-0.42, 0.05; 0.120)	0.96 (0.15)	-0.23 (-0.47, 0.01; 0.059)	0.98 (0.15)	-0.21 (-0.47, 0.04; 0.093)
	TCQ vs. UC			0.09 (-0.19, 0.37; 0.544)		0.03 (-0.25, 0.31; 0.830)		0.24 (-0.05, 0.53; 0.106)		0.01 (-0.16, 0.19; 0.886)

EIM vs. UC	0.08 (-0.21, 0.37; 0.595)	0.06 (-0.23, 0.36; 0.671)	0.08 (-0.22, 0.39; 0.579)	-0.04 (-0.22, 0.15; 0.679)
TCQ vs. EIM	0.01 (-0.24, 0.26; 0.951)	-0.03 (-0.28, 0.21; 0.794)	0.16 (-0.09, 0.40; 0.211)	-0.21 (-0.47, 0.04; 0.093)

Supplemental Table 5. Changes in Sleep from Baseline: Linear Mixed Model Analysis for Within and Between-Arm Comparison for in person sessions

		Baseline	6-week mid-intervention		13 week assessment		3 month assessment		12 month assessment	
				Change from baseline		Change from baseline		Change from baseline		Change from baseline
		Mean (SE)	Mean (SE)	Mean Difference (95% CI; p-value)	Mean (SE)	Mean Difference (95% CI; p-value)	Mean (SE)	Mean Difference (95% CI; p-value)	Mean (SE)	Mean Difference (95% CI; p-value)
PSQI	TCQ (n=30)	5.91 (0.60)	5.47 (0.61)	-0.44 (-1.35, 0.48; 0.348)	5.35 (0.61)	-0.56 (-1.49, 0.37; 0.237)	5.35 (0.61)	-0.56 (-1.49, 0.37; 0.237)	5.97 (0.63)	0.07 (-0.90, 1.03; 0.890)
	EIM (n=22)	7.02 (0.70)	5.82 (0.75)	-1.21 (-2.39, -0.03; 0.045)	6.07 (0.75)	-0.95 (-2.13, 0.23; 0.113)	6.22 (0.75)	-0.80 (-1.98, 0.38; 0.182)	7.33 (0.75)	0.30 (-0.88, 1.49; 0.612)
	UC (n=25)	6.24 (0.65)	5.91 (0.68)	-0.33 (-1.38, 0.72; 0.532)	5.60 (0.70)	-0.64 (-1.72, 0.45; 0.250)	5.30 (0.71)	-0.94 (-2.07, 0.19; 0.102)	5.98 (0.74)	-0.26 (-1.44, 0.92; 0.666)
	TCQ vs. UC			-0.10 (-1.50, 1.29; 0.884)		0.08 (-1.35, 1.51; 0.916)		0.38 (-1.08, 1.84; 0.607)		0.07 (-0.90, 1.03; 0.890)
	EIM vs. UC			-0.87 (-2.45, 0.71; 0.278)		-0.32 (-1.92, 1.29; 0.697)		0.14 (-1.50, 1.77; 0.868)		0.30 (-0.88, 1.49; 0.612)
	TCQ vs EIM			0.77 (-0.73, 2.26; 0.312)		0.39 (-1.11, 1.90; 0.606)		0.24 (-1.26, 1.75; 0.749)		-0.26 (-1.44, 0.92; 0.666)

Supplemental Table 6. Changes in Sleep from Baseline: Linear Mixed Model Analysis for Within and Between-Arm Comparison for mixed

		Baseline	6-week mid-intervention		13 week assessment		3 month assessment		12 month assessment	
				Change from baseline		Change from baseline		Change from baseline		Change from baseline
		Mean (SE)	Mean (SE)	Mean Difference (95% CI; p-value)	Mean (SE)	Mean Difference (95% CI; p-value)	Mean (SE)	Mean Difference (95% CI; p-value)	Mean (SE)	Mean Difference (95% CI; p-value)
PSQI	TCQ (n=14)	6.47 (0.50)	5.77 (0.51)	-0.70 (-1.44, 0.04; 0.065)	5.82 (0.51)	-0.65 (-1.39, 0.08; 0.082)	5.88 (0.52)	-0.59 (-1.34, 0.16; 0.123)	6.24 (0.53)	-0.23 (-1.02, 0.56; 0.569)
	EIM (n=20)	6.69 (0.51)	5.48 (0.55)	-1.20 (-2.02, -0.39; 0.004)	5.86 (0.55)	-0.82 (-1.63, -0.01; 0.048)	5.65 (0.54)	-1.04 (-1.84, -0.24; 0.011)	6.81 (0.55)	0.12 (-0.70, 0.94; 0.770)
	UC (n=25)	6.24 (0.67)	5.91 (0.69)	-0.33 (-1.34, 0.67; 0.514)	5.60 (0.71)	-0.64 (-1.68, 0.40; 0.228)	5.30 (0.72)	-0.94 (-2.02, 0.14; 0.089)	5.99 (0.74)	-0.25 (-1.38, 0.87; 0.658)
	TCQ vs. UC			-0.36 (-1.61, 0.89; 0.567)		-0.02 (-1.29, 1.26; 0.980)		0.35 (-0.97, 1.67; 0.603)		-0.23 (-1.02, 0.56; 0.569)
	EIM vs. UC			-0.87 (-2.16, 0.42; 0.187)		-0.18 (-1.50, 1.14; 0.784)		-0.10 (-1.45, 1.25; 0.882)		0.12 (-0.70, 0.94; 0.770)
	TCQ vs. EIM			0.51 (-0.60, 1.61; 0.367)		0.17 (-0.93, 1.26; 0.764)		0.45 (-0.65, 1.55; 0.421)		-0.25 (-1.38, 0.87; 0.658)
